# Mycotoxins in Pistachios (*Pistacia vera* L.): Methods for Determination, Occurrence, Decontamination

**DOI:** 10.3390/toxins13100682

**Published:** 2021-09-25

**Authors:** Ana Rita Soares Mateus, Sílvia Barros, Angelina Pena, Ana Sanches Silva

**Affiliations:** 1 Faculty of Pharmacy, University of Coimbra, Polo III, Azinhaga de Stª Comba, 3000-548 Coimbra, Portugal; anarsmateus@hotmail.com (A.R.S.M.); ana.silva@iniav.pt (A.S.S.); 2 National Institute for Agricultural and Veterinary Research (INIAV), I.P., Rua dos Lagidos, Lugar da Madalena, 4485-655 Vila do Conde, Portugal; silvia.barros@iniav.pt; 3 LAQV, REQUIMTE, Laboratory of Bromatology and Pharmacognosy, Faculty of Pharmacy, University of Coimbra, Polo III, Azinhaga de Stª Comba, 3000-548 Coimbra, Portugal; 4 Center for Study in Animal Science (CECA), ICETA, University of Oporto, 55142 Oporto, Portugal

**Keywords:** mycotoxins, aflatoxins, pistachios, *Pistacia vera* L., determination, analytical methods, occurrence, decontamination

## Abstract

The consumption of pistachios (*Pistacia vera* L.) has been increasing, given their important benefit to human health. In addition to being an excellent nutritional source, they have been associated with chemical hazards, such as mycotoxins, resulting in fungal contamination and its secondary metabolism. Aflatoxins (AFs) are the most common mycotoxins in pistachio and the most toxic to humans, with hepatotoxic effects. More mycotoxins such as ochratoxin A (OTA), fumonisins (FBs), zearalenone (ZEA) and trichothecenes (T2, HT2 and DON) and emerging mycotoxins have been involved in nuts. Because of the low levels of concentration and the complexity of the matrix, the determination techniques must be very sensitive. The present paper carries out an extensive review of the state of the art of the determination of mycotoxins in pistachios, concerning the trends in analytical methodologies for their determination and the levels detected as a result of its contamination. Screening methods based on immunoassays are useful due to their simplicity and rapid response. Liquid chromatography (LC) is the gold standard with new improvements to enhance accuracy, precision and sensitivity and a lower detection limit. The reduction of *Aspergillus’* and aflatoxins’ contamination is important to minimize the public health risks. While prevention, mostly in pre-harvest, is the most effective and preferable measure to avoid mycotoxin contamination, there is an increased number of decontamination processes which will also be addressed in this review.

## 1. Introduction

Pistachios (*Pistacia vera* L.) are one of the most popular nuts in the world, due to their flavour, nutritional quality and health benefits. Consumption of nuts like hazelnuts, almonds, walnuts, pistachios and cashew nuts is characteristic of the Mediterranean diet [1]. Worldwide, the consumption of pistachios amounted to approximately 761.71 mil tons in 2020. In five years, the consumption increased by approximately 198 mil tons. In United States of America (USA), the per capita consumption of pistachios increased substantially from 0.095 kg in 2015 to 0.245 kg in 2020 [2,3]. The consumption of nuts has been increasing in Portugal; on average, one Portuguese person consumes 6.5 kg of nuts per year [4]. This is in part related with the fact that the consumption of nuts has been associated with a healthy dietary pattern and recommended by health professionals, namely nutritionists, due to pistachios being low in calories, high in mono-unsaturated fatty acids and low in saturated fatty acids. In addition, they are a good source of proteins, carbohydrates, dietary fibers, vitamins (A, E, K, B1 and B6) and minerals (potassium, phosphorus, magnesium and iron). About 100 g of pistachios provides 4 g of the essential amino acid tryptophan [5,6,7]. Pistachio is a very versatile nut, consumed as a snack (raw, roasted, salted or flavored) and also used in ice cream and bakery goods. In 2019, the global market of pistachios was dominated by Iran and the United States of America, which produced 337,000 tons and 335,000 tons, respectively, followed by China and Turkey [8,9].

The composition of nuts is determinant for beneficial effects. From a health point of view, several studies indicate that pistachios reduce the risk of coronary heart disease since there is a reduction in cholesterol levels and a decrease in blood pressure [6]. Other studies suggest a reduction in oxidative and inflammatory stress, blood glucose control, better appetite management and consequent weight control [7].

**Figure 1:**
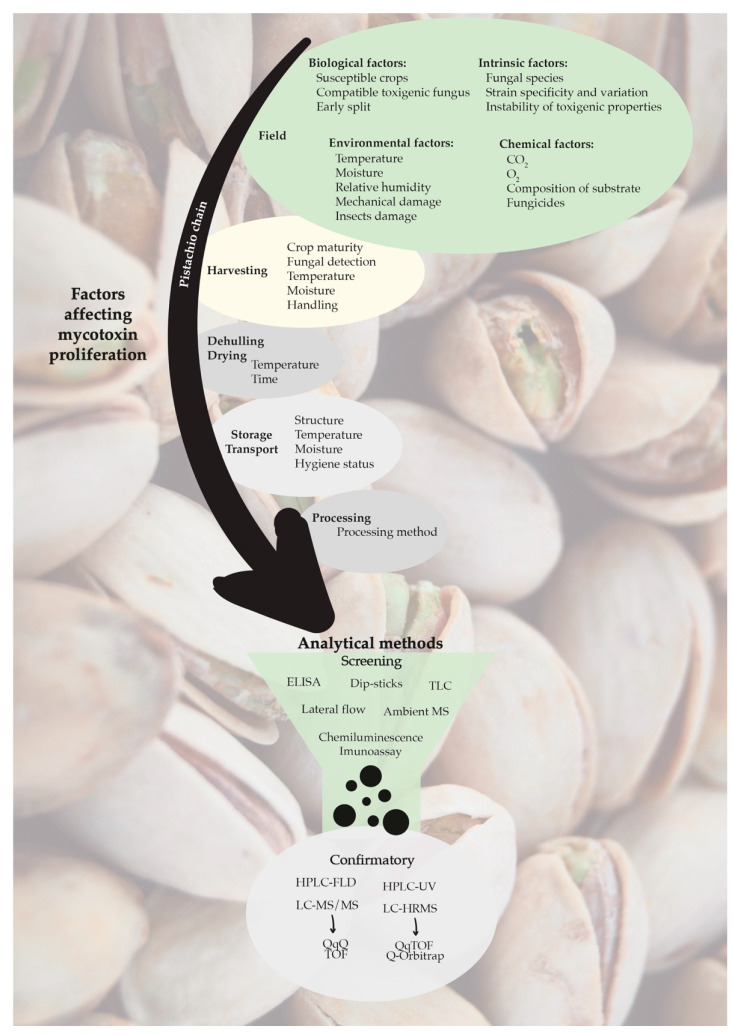
Major factors influencing mycotoxin proliferation along the food chain and main analytical methods for screening and confirmatory determination of mycotoxins in pistachios (ELISA—Enzyme-Linked Immunosorbent Assay; FLD—Fluorescence detector; HPLC—High-Performance Liquid Chromatography; HRMS—High-Resolution Mass Spectrometry; LC—Liquid Chromatography; MS—Mass Spectrometry; MS/MS—Tandem Mass Spectrometry; Q-Orbitrap—Quadrupole-orbital ion trap; QqQ- Triple Quadrupole; QqTOF—Double Quadrupole-TOF; TLC—Thin-layer chromatography; TOF—Time-of-flight).

Similar to other nuts, pistachio contains low amounts of water after being dried, which restricts spoilage by microorganisms. However, some fungi are able to develop, since they require a smaller amount of water to multiply [10]. Fungal contamination can occur along the food chain, in the development of the plant in the field, as well as in post-harvest, drying, transport, storage and processing. Contamination may occur in these phases following harvest or there may be an increase in previous contamination [11]. Fungal contamination is closely related to environmental conditions, such as temperature and humidity, which must be favorable to its growth. Moreover, crop damage due to insect infestation and improper drying of crops before storage are factors to be taken into account [12] (Figure 1).

As a result of this contamination, mycotoxins appear in nuts. The word “mycotoxin” is derived from the Greek word “mykes” meaning “fungus” and the Latin word “toxicum” meaning “poison” [13]. Mycotoxins are secondary metabolites of filamentous fungi; low mass molecules produced by multiples genera and species of fungi have in common toxic effects in animals and humans. Mycotoxins are a heterogeneous group due to several chemical structures, biosynthetic origins and biological effects [14]. Food may be contaminated with several different mycotoxins because, when conditions are favorable for fungal contamination, more than one fungal species can contaminate food, and also, a single species of fungi can produce several toxic metabolites [11,15]. It is also important to mention that the presence of fungi may not be related to the presence of mycotoxins. On the one hand, not all fungi are mycotoxin producers, and on other hand, mycotoxins are only produced under certain conditions. In fact, the occurrence of aflatoxin contamination is sporadic and, although large populations of *A. flavus* infect crops, serious outbreaks are associated with above-average temperature and below-average rainfall [11].

Mycotoxins have different adverse effects on human health, such as, carcinogenicity, mutagenicity, teratogenicity, cytotoxicity, neurotoxicity, nephrotoxicity, immunosuppression and estrogenic effects [16]. The severity of the effects depend on amounts ingested, duration of exposure and on individual characteristics, such as age, gender, weight, diet or health status [17]; for example, a low variety and insufficient diet constitute a risk factor for greater severity of negative effects of mycotoxicosis [18]. In addition, the interaction between mycotoxins could result in antagonistic, additive or synergistic effects [19,20].

Mycotoxins are a concern for food safety, Food and Agriculture Organization of the United Nations (FAO) estimates that 25% of foods are contaminated by mycotoxins, with consequences on health but also leading to economic losses at all levels of the food chain [11]. Mycotoxins are more common in developing countries, where less concern for food safety, insufficient quality control, hot weather, inadequate production techniques and poor crop storage conditions are suitable for the growth of fungi [21]. However, contamination is a global concern because it is an unpredictable and inevitable problem, one of the most challenging to food safety, even when all good practices in the food chain are implemented. The Rapid Alert System for Food and Feed (RASFF), in 2018, reported 569 notifications for mycotoxins, predominantly in the group of dried fruits, derived from dried fruit and seeds, such as nuts, pistachios and almonds. The most prevalent reported group are aflatoxins, followed by ochratoxin A. The same trend is maintained in 2019, with 588 notifications for mycotoxins and 90% of notifications are from countries outside the EU, particularly, Turkey and Argentina. In pistachio nuts, RASFF, between January 2020 and June 2021, reported 84 notifications, mostly from Turkey, Iran and the USA, related with aflatoxins and one notification concerning ochratoxin A in pistachios.

To ensure consumer health, the occurrence of mycotoxins is monitored, and maximum levels are regulated worldwide. In the European Union, the European Food Safety Authority (EFSA) is responsible for scientific opinions concerning risks associated with mycotoxins and advice to the European Commission (CE), which established Regulation no. 1881/2006 concerning the maximum levels of certain contaminants, including certain mycotoxins. The levels of aflatoxins in foodstuffs not for direct human consumption are higher as they will still be processed. Based on the toxicity of different aflatoxins, a limit is provided for the total aflatoxins in food, corresponding to the sum of aflatoxin B1 (AFB1), aflatoxin B2 (AFB2), aflatoxin G1 (AFG1) and aflatoxin G2 (AFG2), as well as the individual content of AFB1 since this is aflatoxin with the greatest concern given its carcinogenicity. Peanuts and nuts available on the market for the consumer must have a content of AFB1 less than 2 μg/kg and total aflatoxin content of less than 4 μg/kg. As aflatoxins (AFs) are carcinogenic substances, maximum levels should be imposed at a level that is as low as reasonably achievable (ALARA), defined as “the concentration of a substance that cannot be eliminated without seriously compromising the availability of main food nutrients” [11]. In Codex Alimentarius, maximum levels for total aflatoxins in treenuts, including almonds, hazelnuts, pistachios and shelled Brazil nuts, for human direct consumption are 10 μg/kg and for treenuts still to undergo further processing are 15 μg/kg. However, maximum levels of Desoxynivalenol (DON), fumonisins (FB1 and FB2) and ochratoxin A (OTA) in nuts are not established [22]. EFSA [23] published a scientific opinion concluding that increasing the maximum level of AFs in pistachios, almonds and hazelnuts to 8 or 10 μg/kg would increase aflatoxin exposure by 1%, with more impact in groups with a high level of nut consumption, and, despite the minor effects on cancer risk, EFSA strengthens the recommendation that exposure to AFs should be as low as reasonably achievable. European legislation covers other mycotoxins, for example, OTA, but in dried fruit other than raisins, the maximum levels are not defined yet, and for DON, zearalenone (ZEA), FB1, FB2, and toxins T-2 (T2) and H-T2 there is no reference to the maximum levels in nuts. 

Thus, pistachios, despite their beneficial effects on human health, also have chemical hazards and are an important source of exposure to mycotoxins, especially aflatoxins, constituting a current public health problem. Pistachios are considered to be the ones with the highest risk of contamination by aflatoxins, largely due to shell splitting at end of maturation [24,25]. This shell protects the pistachio kernel and, as a consequence of splitting, pistachios are susceptible to molds and insect invasions. For example, navel orangeworm (NOW) (*Amyelois transitella*) is a common pest of pistachio nuts in the field. This worm causes direct physical damage in pistachios due to the worm’s growth, feeding on kernels and insect excrement [26]. However, it also causes indirect damage because it predisposes contamination by the aflatoxin-producing fungi. In fact, a study focused on California pistachios showed that kernel infested by NOW had substantially more infections by *Aspergillus* fungi producers of AFs and OTA, *A. flavus* and *A. niger*, respectively, and consequently AFs are more frequently found in higher levels [27].

The present paper is the result of a vast literature review performed to evaluate the state of the art of the determination of mycotoxins in pistachios concerning the new trends in analytical methodologies for its determination and the levels detected as a result of its contamination. Moreover, the mitigation strategies are presented, namely regarding the decontamination by physical, chemical or biological methods.

## 2. Aflatoxins

Aflatoxins (AFs) are a class of mycotoxins produced by fungi of the genus *Aspergillus*, especially the species *A. flavus* and *A. parasiticus.* Fungi *A. nomius*, *A. pseudotamari*, *A. bombycis* and *A. ochraceoroseus* are also producers of aflatoxins but found less frequently [28]. *Aspergillus* are distributed worldwide, but the great predominance is in countries with subtropical climate and warm temperature. They are characteristically greenish to greyish molds and grow in hot (15 to 40 °C) and humid conditions. 

### 2.1. Physical and Chemical Characteristics

**Figure 2:**
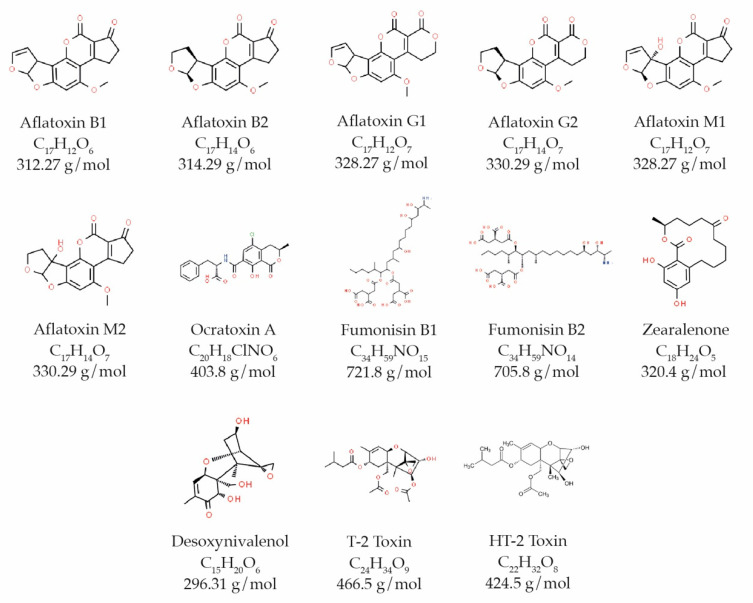
Examples of the main mycotoxins’ most common determinate in foods (structures from www.chemspider.com (accessed on 11 May 2021)).

Aflatoxins are low molecular weight molecules, among 312–346 Da [29], composed of carbon, oxygen and hydrogen atoms (Figure 2). They are highly oxygenated heterocyclic compounds derived from difuranocumarinic, where the difuran group is attached to one side of the cumarin nucleus and the pentatone ring is connected to the other side, in the case of AF-B series, or the hexagonal lactone ring, in the case of AF-G series [28]. The designation of series is related to fluorescence of molecules under UV light: B series has a blue color and G series has a green color, while associated numbers are related to the mobility of molecules in chromatography [11]. 

More than 20 aflatoxins are known, but the four main ones are: Aflatoxin B1 (AFB1), aflatoxin B2 (AFB2), aflatoxin G1 (AFG1) and aflatoxin G2 (AFG2), as well as the metabolites of AFB1 and AFB2, aflatoxin M1 (AFM1) and M2 (AFM2), respectively, as they were primarily found in animal milk [14]. It should be noted that *A. flavus*, more common in dried fruits [28], mainly produces B-series aflatoxins, while *A. parasiticus* produces both aflatoxins B and G [30]. In terms of toxicity, the most toxic aflatoxin is AFB1, followed by AFG1, AFB2 and, the least toxic, AFG2 [31], while AFM1 has similar toxicity to AFG1 [32].

These mycotoxins are characterized by being crystals that are colorless to light yellow. They present fluorescence under UV light, but UV light is instable in the presence of extreme oxygen and pH (<3 or >10). The melting points of these molecules are between 240 and 280 °C. AFs are soluble in organic solvents, such as chloroform and methanol, moderately soluble in water and insoluble in non-polar solvents [31].

### 2.2. Toxicokinetics

AFB1 is the best studied aflatoxin due to its relevance in human health and to its being the one that most frequently occurs in food, reflecting metabolisms of other AFs. AFB1 is rapidly absorbed by the gastrointestinal tract, reaching maximum concentrations in the bloodstream after 1 h [33]. About 95% of AFB1 and metabolites are excreted in the urine in the first 24 h after exposure [33]. AFB1 is metabolized in the liver by the cytochrome P450 system, by epoxidation, to an electrophilic and very reactive molecule, aflatoxin B1-exo-8,9-epoxide (AFBO), capable of covalently binding to DNA, RNA and proteins [14]. Conjugation of AFBO with glutathione by glutathione-S-transferase is a detoxification route since it inhibits the ability of AFBO to bind to DNA, forming an inert metabolite, followed by biotransformation with mercapturic acid, and then excreted in urine [34]. In addition to epoxidation, AFB1 can be metabolized by hydroxylation reaction and also by cytochrome P450 system enzymes, resulting in several metabolites: Aflatoxin M1 (AFM1), aflatoxin Q1 (AFQ1), aflatoxin P1 (AFP1), aflatoxicol (AFL), aflatoxicol H1 (AFH1) and aflatoxin B2a (AFB2a). AFM1 is the predominant metabolite, most commonly found as a consequence of AFB1 exposure, and the most carcinogenic, by a similar mechanism concerning AFB1. Moreover, these metabolites have toxic effects on humans [34]. AFM1 and AFQ1, although toxic, are less reactive than other molecules and are eliminated directly in urine [35].

### 2.3. Toxicity 

Aflatoxins are the leading cause of non-infectious diseases of food origin. It is estimated that 4.5 to 5.5 billion people are exposed to these mycotoxins [36]. AFs are genotoxic, carcinogenic and hepatotoxic; therefore, there is no threshold level for their toxicity and a tolerable daily intake is not established. The Joint FAO/WHO Expert Committee on Food Additives (JECFA), in 1997, through epidemiological data, estimated that intake of 1 ng AFB1/kg bw/day increases the incidence of liver cancer by 0.013 cancer cases/year per 100 000 subjects, for HBsAg-negative individuals, concerning risk assessment. In 2016, JECFA recalculated the cancer risk associated with aflatoxin exposure and concluded that European people and those of other developed countries had a lower cancer risk, ranging from <0.01 to 0.1 aflatoxin-induced cancers per year and per 100,000 subjects [37].

#### 2.3.1. Acute Toxicity

Exposure to high concentrations of aflatoxins in a short period of time leads to hepatotoxic effects, manifesting early as anorexia, malaise and low fever, and maybe progressing to vomiting, abdominal pain and jaundice, as well as pulmonary and cerebral edema, coma and convulsions [38]. In addition, acute exposure to a high AF content can lead to death by hepatitis [28]. Estimated total aflatoxin intake that causes a mortality risk is >1 mg/day, i.e., >20 μg/kg body weight/day in adults [33]. Children are a more vulnerable population group since consumption of food by body weight is higher compared to adults; immune and neurological systems are immature and diet is more restricted, so there is greater susceptibility to development of complications [18]. AFB1 may cause weight loss, growth delay or even malnutrition states in children [39]. Acute exposure to AFs is associated with Kwashiorkor Syndrome, identified through epidemiological studies and outbreaks that have occurred throughout history. Kwashiorkor syndrome is intermediate malnutrition associated with high carbohydrate intake due to a lack of proteins and vitamins and occurs mainly in children. Studies indicate that children with this syndrome are more exposed to AFB1 by cereals consumed and have a higher frequency and higher concentration of aflatoxicol in serum, indicating a change in AFB1 metabolism and interference in micronutrient absorption [34]. Some studies also indicate a relationship with Reye Syndrome, an acute encephalopathy with visceral fat degeneration, more common in adolescents; however, the cause–effect relationship of aflatoxins with this syndrome has not yet been fully established [11].

#### 2.3.2. Chronic Toxicity

Aflatoxin B1 is considered the most potent hepatic carcinogenic of aflatoxins, and the International Agency for Research on Cancer (IARC) since 1987 classified it in group 1, proven carcinogenic to humans, related to hepatocellular carcinomas, since there is sufficient scientific evidence in both studies conducted on animals and human studies [40]. Toxicity mechanisms are related to the metabolite of AFB1. AFBO is capable of linking to DNA, by nucleophilic addition, to nitrogen 7 (N7) of guanine base, forming AFB1-N7-guanine adduct. The formation of this adduct in DNA leads to G-to-T transversion during cell replication. One consequence is the AGG–AGT (Arginine–Serina) transversion, resulting in the inactivation of the p53 tumor suppressor gene in codon 249, responsible for cell cycle control, DNA repair and apoptosis [11,35]. In addition, AFBO can bind to primary amine groups of amino acids (such as lysine) and proteins (namely albumin), forming adducts found in the bloodstream [14,35].

For epidemiological studies, it was concluded that exposure to AFs constitutes a risk factor for the development of hepatocellular carcinoma (HCC) [14]. HCC is the fourth most common cause of cancer-related death worldwide. In addition to exposure to AFs, alcohol, hepatitis B and C and other metabolic liver diseases are considered risk factors for HCC [41]. Epidemiological studies conducted in Asia and Africa have indicated a combination of AFB1 exposure and hepatitis B virus (HBV) infection increases the risk of HCC; that is, there is a synergistic effect between AFB1 and HBV. The first clinical evidence of this synergism occurred in China where it was found that HCC occurred in individuals infected with HBV living in villages with high consumption of aflatoxins, with a mortality rate 10 times higher than in individuals living in villages with lower consumption [36]. HBV infection can sensitize hepatocytes to carcinogenic effects of AFB1, explained by different mechanisms related to mutation in codon 249. One hypothesis states that the HBV genome is inserted in the HBV X gene, translated into the HBV X protein that inhibits DNA repair and also contributes to uncontrolled cell proliferation. Another hypothesis states that necrosis of hepatocytes and proliferation results in an increase of cells with mutation. Moreover, chronic inflammatory liver disease, resulting from the HBV virus, causes production of reactive oxygen and nitrogen species that increase oxidative stress and can induce mutation [36]. In addition, these studies have shown that exposure to AFB1 alone was sufficient to significantly increase the risk of developing cancer [34]. Hepatitis C virus (HCV) has also shown a correlation with the incidence of HCC, in synergy with exposure to AFB1, but this is not yet fully established [34].

Children are chronically exposed to high levels of aflatoxins in areas where food contamination is endemic, and this exposure begins in the uterine phase, in the fetal development, through mother’s milk, and continues throughout life [33]. AFs are considered a risk factor for compromising children’s growth [34]. Furthermore, studies show that AFB1 has the ability to decrease immune system functions, with changes in immunological parameters in populations chronically exposed to aflatoxins [33,34].

## 3. Ochratoxin A (OTA)

Ochratoxin A (OTA) is the second most important mycotoxin produced by fungi *Aspergillus ochraceus*, *A. carbonarius* and *Penicillium verrucosum*. This occurs predominantly in cereals and derivatives, namely flours, bread, rice, breakfast cereals and infant feed [11]. OTA have nephrotoxic effects associated with oxidative stress. In humans, epidemiological studies demonstrate a possible association with Balkan endemic nephropathy and endemic chronic interstitial nephropathy, but a causal link has not yet been established [38,42]. It is classified by the IARC as possibly carcinogenic to humans and belongs to group 2B since there is sufficient scientific evidence of carcinogenicity in animals, but human studies are still insufficient [33,40]. Moreover, OTA is considered immunotoxic, neurotoxic, mutagenic, teratogenic and hepatotoxic and affects development [39,42]. In 2008, JECFA reconfirmed a provisional tolerable weekly intake (PTWI) of 100 ng OTA/kg bw from 1995, and estimated that dietary exposures, mainly in Europe, ranging from 8 to 17 ng/kg bw per week are below the PTWI [42].

OTA is a polypeptide derivative of dihydro-isocomarina, bound by the 7-carboxylic group to 1-b-phenylalanine by an amide bond (Figure 2). Characterized by being a white crystal with a melting point of 90 °C, when recrystallized with benzene, it is very soluble in polar organic solvents, moderately soluble in water and soluble in sodium hydrogencarbonate solutions. It presents absorption in ultraviolet to λ_MeOHmax_ (nm; ɛ) = 333 (6400) and intense native fluorescence, with a maximum emission at 467 nm in 96% ethanol [43,44].

## 4. Fumonisins (FB1 and FB2)

Fumonisins are produced by the fungi *Fusarium proliferatum* and *F. verticillioides*, predominantly found in corn and derived products. Fumonisin B1 (FB1) is the most toxic fumonisin, followed by fumonisin B2 (FB2) [45]. However, it has recently been discovered that *Aspergillus niger* also produces FB2 [17].

Fumonisins are characterized by a long chain hydroxylated hydrocarbon, hydroxyl groups in C14 and C15 esterified with terminal carboxylic group of tricarboxylic acid (Figure 2) [17]. They are different molecules from other mycotoxins because they are hydrophilic, dissolve completely in organic solvents such as methanol and acetonitrile:water (1:1) and do not present fluorescence [14,46]. FB1 and FB2 are structurally similar to sphingosine and sphinganin bases. They interfere with the metabolism of sphingolipids, competitively inhibiting the ceramide synthase enzyme, causing dysregulation in cell cycle [17,38,47]. These mycotoxins are considered to be possibly carcinogenic to humans, belonging to IARC Group 2B. They are associated with esophageal cancer [48]. The largest target organs of these mycotoxins are the liver and the kidneys, and FB1 is carcinogenic, hepatotoxic and nephrotoxic [38,48]. JECFA (2011) [49] established a provisional maximum tolerable daily intake (PMTDI) for FB1, FB2 and FB3 of 0.002 mg/kg bw, alone or in combination.

## 5. Zearalenone (ZEA)

Zearalenone (ZEA) is a secondary metabolite of fungi of the genus *Fusarium*, mainly of the species *F. graminearum* and *F. culmorum* [47], very common in cereals such as corn, wheat, barley, rye and their derivatives [38].

This mycotoxin is a macrocyclic–resorcyclic acid lactone (Figure 2) [38], with a similar structure to 17-β-estradiol, a human sex hormone, so ZEA is considered a non-steroidal estrogenic mycotoxin [38,46,47]. Given this structural similarity, they have affinity for estrogen receptors and, as a consequence, lead to negative effects on the reproductive system, such as fertility problems, precocious puberty, change in serum levels of estradiol and progesterone [17,45]. IARC categorized ZEA in group 3, not classified as carcinogenic to humans, since studies are limited [40]. In 2000, JECFA established a provisional maximum tolerable daily intake (PMTDI) for ZEA of 0.5 μg/kg/bw.

ZEA presents in the form of white crystals, is soluble in benzene, acetonitrile, methanol, ethanol and acetone, is very stable for degradation up to 120 °C and is stable to hydrolysis in neutral or acid buffer solutions.

## 6. Trichothecenes

Trichothecenes are a group of structurally related mycotoxins produced mainly by fungi of the genus *Fusarium*. These molecules consist of a 12,13-epoxytrichothene skeleton and a double bond with several substitutions in the side chain (Figure 2). This group includes non-macrocyclic mycotoxins: Desoxynivalenol (DON), T2 toxin and HT-2 toxin [14], all classified in group 3 of IARC, due to inadequate scientific evidence in animals and a lack of human studies [40]. These mycotoxins are cytotoxic, interfering in synthesis of nucleotide acids and proteins and cell division [17].

### 6.1. Desoxynivalenol (DON)

Desoxynivalenol is a B-type trichothecene with carbonyl group in carbon 8 (Figure 2) [14]. Mainly produced by the species *Fusarium graminearum* and *F. culmorum*, it is very common in cereals such as wheat and corn [45]. DON is known as vomitoxin, due to its acute exposure and is linked to gastroenteritis in humans with nausea, vomiting, abdominal pain, headache, fever and also with immunosuppressive effects, mostly reported in Asia [50]. They deregulate the normal functioning of cells, by inhibiting protein synthesis, influence on signaling, differentiation and cell proliferation [51]. In 2011, JECFA established PMTDI for DON and its acetylated derivatives (3-Ac-DON and 15-Ac-DON) of 1 mg/kg bw/day, and also established an acute reference dose (ARfD) of 8 mg/kg bw [50]. DON was later recognized as responsible for an epidemic in Japan called “red mold poisoning” due to consumption of maize and moldy wheat, whose symptoms were nausea, vomiting, diarrhea and seizures [30]. DON is characterized by white needle-shaped crystals. It is soluble in chloroform, ethanol, methanol and ethyl acetate and stable at pH 4 even at high temperatures [46].

### 6.2. HT-2 Toxin and T-2 Toxin

HT-2 and T-2 toxins are A-type trichothecene, with a hydrogen or an ester group in lateral chain in carbon-8; the difference between these two molecules is the carbon-4-bound group: In the case of HT-2 it is a hydroxyl group, and in the case of T2 it is an acetate group (Figure 2) [14]. These mycotoxins are produced by species *Fusarium sporotrichioides* and *Fusarium poae*, found especially in oats and also in corn and wheat [45]. HT-2 toxin (HT2) is a metabolite of T-2 toxin (T2). T-2 toxin has a haematotoxicity effect and is linked to food toxic aleukia (ATA), a condition that involves irritation of gastrointestinal tract, vomiting, diarrhea and, in the most severe cases, leukemia, anemia and even death [14,17]. Some in vivo studies show that T2 and HT2 have anorectic effects upon short-term exposure [52]. In 2016, EFSA 2016 established a tolerable daily intake (TDI) for T2 and HT2 of 0.02 mg/kg bw/day based on immune- and haematotoxicity of T2. The EFSA scientific report [53] shows a high chronic exposure in lower age groups.

## 7. Emerging mycotoxins

Besides common mycotoxins, there is also a group of emerging mycotoxins, defined as “mycotoxins, which are neither routinely determined, nor legislatively regulated; however, the evidence of their incidence is rapidly increasing” [54]. These new mycotoxins are more usually found in cereals like wheat, maize and barley, and Mediterranean crops; determination on pistachios and other tree nuts are rare.

*Fusarium* second metabolites like fusaproliferin (FUS), beauvericin (BEA), enniatins (ENNs), and moniliformin (MON) are included in the group of emerging mycotoxins. Moreover, fusaric acid, culmorin, butanolide [55] and, more recently, NX-2 [56] are *Fusarium* emerging mycotoxins. Moniliformin (MON) was first described by Cole et al. [57] isolated from the *Fusarium* strain, initially called *F. moniliforme*, which contaminated cereals like maize. MON is a small, water-soluble and very acidic molecule that occurs in nature typically as sodium or potassium salt [58]. The toxicity of MON is due to the inhibition of thiamine enzymes, compromising the tricarboxylic acid cycle and resulting in cytotoxic effects for lymphocytes and cardiomyocytes. Muscle weakness, breathing difficulties and myocardial lesions are reported symptoms resulting from MON exposure, based on animal studies, and the heart is the main target organ [59]. However, MON is suspected to be associated with the development of Keshan’s disease, an endemic disease reported in China characterized by myocardial insufficiency [55,58].

Beauvericin (BEA) and Enniatins (ENNs) are structurally very similar mycotoxins found in grains and cereal based food. *Fusarium* species like *F. proliferatum*, *F. subglutinans* or *F. verticillioides* produce BEA, primarily found in 1969, and *F. avenaceum*, *F. poae* or *F. tricinctum* produce ENNs, and ENN A, A1, B and B1 are the most commonly detected in food. *F. oxysporum* produces both mycotoxins. The toxicity of BEA and ENNs are based on their ionophore proprieties; they act as transporters for mono- or divalent cations, for example, K^+^ or Ca^2+^, resulting in disruption of normal physiological concentrations, inducing DNA fragmentation and apoptosis. BEA and ENNs have also been demonstrated to inhibit acyl-CoA:cholesterol acyltransferase (ACAT) which causes the accumulation of cholesteryl ester in atherogenesis. BEA and ENNs have no cytotoxic in vitro studies and no mutagenicity in the Ames test. Moreover, they show pharmacological properties, such as anticonvulsant, antineoplastic and lower cholesterol levels of blood [58]. EFSA (2014) conclude that acute exposure to BEA and ENNs is not a concern to human health and since there is a lack of toxicity in in vivo data, there are no conclusions concerning chronic exposure [55,58]. Liao et al. [60] detect 1.9 μg/kg of BEA in one sample of roasted pistachios, out of a total of ten samples.

Fusaproliferin (FUS) is one of the most recent mycotoxins, discovered in 1993 by Randazzo et al., so very little is known about it yet. Most of the studies are in plants, insects, and cell cultures. These studies indicate that FUS have phytotoxic properties, are moderately cytotoxic to human B lymphocyte, interact with DNA and show teratogenic effects [55,58]. However, toxicity and mode of action have not been comprehensively investigated and there is still an insufficient amount of toxicity data to assess the impact on human health.

In addition *Aspergillus*, *Alternaria* and *Penicillium* are fungi that produce emerging mycotoxins. Sterigmatocystin (STC) is an *Aspergillus* mycotoxin, mainly produced by *A. nidulans* and *A. versicolor*, and a structurally closely related and toxic precursor to aflatoxins [55]. Studies show mutagenicity and cytotoxic effects, with formation of DNA adducts. In 1987, IARC classified STC in group 2B (possibly carcinogenic to humans) [61]. *Alternaria* mycotoxins are mostly produced by *Alternaria alternata* and include alternariol, alternariol monomethyl ether, tenuazonic acid (TeA) and altertoxins with some effects in animals.

## 8. Analytical Methods for the Determination of Mycotoxins

Mycotoxins are present in low concentrations, in the order of μg/kg, and nuts represent a complex food matrix, mainly due to the lipid content (53%) [5,62]. Therefore, sensitive analytical methods with low limits of detection and quantification and good specificity, precision and accuracy are needed [63]. Analysis of mycotoxins, regardless of analytical method, follows a common protocol: Sampling, sample preparation, extraction, with or without purification, and detection/quantification [64]. Sample preparation is very important because this step is responsible for eliminating matrix interferents and pre-concentrate mycotoxins and transferring them to an adequate solvent for the next analytical technique [65]. High-performance liquid chromatography (HPLC) or ultra-high pressure liquid chromatography (UHPLC) with fluorescence detection (FLD), mass spectrometry (MS) or tandem mass spectrometry (MS/MS) are the main analytical techniques reported in the scientific literature. Other researchers have more recently used immunoassays, like enzyme-linked immunosorbent assay (ELISA) and sensor methodology. Analytical methods used for mycotoxin determination in pistachio and other related food matrices are summarized in Table 1. The most used analytical techniques for screening and confirmatory determination of mycotoxins in pistachio are represented in Figure 1. In screening analysis, immunoassays are the most applied techniques, due to simple and rapid performance; and for confirmatory analysis, liquid chromatography is the gold standard, with distinct detectors. The validation of the analytical methodology, whether it is for screening or confirmatory, is of utmost importance in order to assure reliable data.

Mycotoxins are distributed heterogeneously and may only occur in a fraction of samples [79]. Thus, sampling and preparation of a sample are crucial steps in the determination of these chemical contaminants, to ensure representativeness.

For the determination of AFs in treenuts “ready-to-eat”, Codex Alimentarius recommend a sample of 10 kg of pistachio in-shell nuts or 5 kg shelled nuts, and the sample should be finely ground and mixed thoroughly using a process, to reduce particular size and disperse the contaminated particles evenly throughout the sample, ensuring homogenization, since distribution of aflatoxin and other mycotoxins is extremely non-homogeneous [22]. During sample preparation, it is important to keep samples away from sunlight and also control temperature and humidity in order to not favor mold growth and aflatoxin formation [22]. Pre-treatment of a sample is considered a fundamental and indispensable step in almost all analytical procedures, especially for analysis in complex food matrices [15].

In Europe, sampling and analysis methods for the official control of the mycotoxins in food are established in Regulation No. 401/2006. To analyze AFB1 and AFs in pistachios an overall sample of 30 kg is recommended, resulting from 10 to 100 elementary samples collected from different points of one lot, depending on the lot’s weight. This sample is mixed and divided into two or three equal samples for a laboratory with ≤10 kg before crushing. Then, each laboratory sample is separately finely ground and carefully mixed to ensure complete homogenization. In the case of lots in retail packaging, each package could be considered as one sample for analysis when it is less than 300 g.

The first step in sample preparation is extracting mycotoxins from the solid matrix to a liquid phase, separating them from other components. The extraction solvent is a mixture of an organic solvent with water, where the presence of water favors penetration of organic solvents into a matrix, and, in some cases, acids are used to break the bond of mycotoxins to other components, increasing the effectiveness of extraction [15]. The extraction solvent is chosen according to the characteristics of mycotoxins and matrices [80], and acetonitrile (ACN) is the organic solvent extraction more applied, followed by methanol (MeOH). Moreover, sodium chloride (NaCl) and n-hexane are usually added, in addition to solvent methanol:water [70], due to the higher fat content of pistachio.

The second step is a clean-up to remove interferers and impurities from the extract, such as lipids, proteins and other small molecules, to ensure sensitivity and selectivity. Solid–liquid extraction techniques are often used, namely solid phase extraction (SPE), solid phase micro-extraction (SPME) and solid phase matrix dispersion (MSPD). However, in the pistachio nuts, researchers use immunoaffinity chromatographic columns (IACs) and the QuEChERS (Quick, Easy, Cheap, Effective, Rugged and Safe) method.

IAC is a very sensitive and selective technique because specific antibodies are used for mycotoxins. Affinity of antibody and reversibility of binding are very important because the aflatoxin–antibody complex has to be dissociated to release mycotoxins in the elution phase. The complex has to be stable enough for the washing steps [81]. In a simplified way, the sample is applied into a column with anti-mycotoxin antibodies; then, the column washes, and the final step is the elution of mycotoxins. The eluate is evaporated until dryness to reduce volume and concentrate mycotoxin in the extract. Finally, the residue is redissolved into the mobile phase to follow chromatography analysis [82].

The QuEChERS method, in a simplified way, is divided into two extraction stages. The first extraction step is based on the salting-out effect, with an organic phase in the presence of salts for extraction. Acetonitrile (ACN) is the most used extraction solvent, applicable to a wide range of organic compounds, without co-extraction of interferent molecules from the matrix [83] and easily parts from water in the second phase [84]. To increase efficiency, acidification with formic acid (FA) [62,71,75], acetic acid [85,86] or citric acid [87] is frequently applied. In the case of mycotoxins, a combination of magnesium sulfate (MgSO_4_) with sodium chloride (NaCl) in a 4:1 ratio is the most applied extraction salt. Magnesium sulfate allows the best salting-out of ACN and the best overall recoveries especially of polar analytes; however, MgSO4 contributes to the remaining parts of water in the acetonitrilic layer, so it helps to control the polarity of the extraction solvents and thus increases the selectivity of extraction [88]. In a second phase, the extract is cleaned with adsorbents to remove interferers. Generally, dispersive Solid Phase Extraction (d-SPE) is applied with primary secondary amine (PSA), octadecyl silica (C18) or graphitized carbon black (GCB) [84]. More recently, new adsorbents have been available on the market, for example, EMR-Lipid and Z-Sep^+^. Alcántara-Dúran et al. [75] compare two adsorbents: (1) EMR-lipid, remove lipids based on hydrophobic interactions and exclusion by size between long aliphatic chains of lipids and adsorbent [84]; (2) PSA, which is useful for removing lipids, namely fatty acids, sugars, organic acids and some pigments; and (3) C18, which is recommended for the removal of high lipid content [84]. The authors concluded that EMR-lipid presented the best results, with better percentage of recovery and lower matrix effect. Cunha et al. [62] performed a clean-up with Z-sep^+^ and C18. Z-sep^+^ is composed of C18 and zirconia oxide bound to the same silica particle, removing fatty acids and pigments [84]. Some authors select immunoaffinity chromatography for sample cleaning [85,86].

QuEChERS has numerous advantages like reduction of the steps; simple and easy implementation; separation of a wide range of analytes and several samples in a short time; and use of a smaller volume of samples and solvents, according to the principles of green chemistry [15,62,84]. In addition, QuEChERS is also used in multiclass analysis with simultaneous analysis of multi-mycotoxin and multi-pesticide residues, for example, in cereals [89,90,91].

### 8.1. Detection and Quantification

#### 8.1.1. Chromatographic Techniques

Chromatographic methods are based on the physical interaction between a mobile and a stationary phase. Analytes are differently distributed between two phases, depending on their characteristics, resulting in different speed movements in the column, causing separation [35]. Thin-layer chromatography (TLC), gas chromatography (GC) and liquid chromatography (LC) are used for analysis of mycotoxins. TLC is more used for specific identification of mycotoxins. GC was abandoned because it needs a derivatization step due to most mycotoxins being nonvolatile and polar substances.

In the case of confirmatory identity and quantitative determination of mycotoxins, namely in nuts, liquid chromatography (LC) is the most common technique, given its high precision, high sensitivity and low detection limit [65]. While reversed-phase elution and C18 columns are mostly used, LC mycotoxin analysis is a flexible technique; it can use different elution modes, different column sizes, different particular sizes and different mobile phase compositions in order to improve mycotoxin separation. In recent years, different approaches have been applied to LC mycotoxin analysis, improving efficiency and resolution, making it faster and cheaper. For example, the reduction of particle size or column diameter results in ultra-high liquid pressure chromatography (UHPLC) and capillary/nano-LC, respectively. Moreover, coupling two or more separation columns or using enrichment/extraction first column to online sample preparation are new strategies [65].

Previously, LC was combined with ultraviolet-visible detector (UV-Vis) and fluorescence detector (FLD) for AF analysis due to their fluorescent properties (AFB1 and AFB2 exhibit fluorescence at 425 nm, AFG1 and AFG2 exhibit fluorescence at 450 nm); however, quenching occurred due to the mobile phase, hindering detection of AFs at lower concentrations, requiring derivatization [92]. For AF determination with FLD, the derivatization step (pre- or post-column) is needed to promote sensitivity and resolution. Chemical derivatizations involve chemical reaction between AF and acid (trifluoroacetic acid) or halogen (bromine or iodine) molecules to improve fluorescence. Photochemical derivatization is based on derivatization of AF with UV radiation generated by a photochemical reactor, and there is no need to add any chemical reagents, which is more advantageous [15,35,92]. Photochemical derivatization is the most reported PCD for the determination of AFs in pistachios [68,70,72,74], although some previous studies use bromination [24,69]. However, this derivatization step, especially with chemical derivatization, added complexity to analysis. In addition, other mycotoxins do not have these fluorescence proprieties, so this detection method is not suitable for multi-mycotoxin determination.

More recently, mass spectrometry (MS) was coupled as a detector, resulting in LC-MS based on a separation of analytes by LC and subsequent analysis of mass to charge (m/z) of ions in the gas phase, obtaining structural information that identifies molecules based on molecular weight [92,93]. Nowadays, LC-MS is the most suitable technique recommended by the guidelines for identification, quantification and confirmation of multi-class mycotoxins, being highly sensitive and specific and one of the best options for this type of analytical determination in complex food matrices [92]. LC-tandem mass spectrometry (LC-MS/MS) is a powerful technique for mycotoxins because of its ability to detect multiple regulated, unregulated and emerging mycotoxins, with a need of precursor ions to correct identification and quantification [65]. LC-MS can be performed employing different MS analyzers to increase detection abilities, and provide different information and data treatment and emerging LC-High Resolution Mass Spectrometry (HRMS). For example, there are classical, like triple quadrupole (QqQ) and time-of-flight (TOF), or hybrid modern detectors such as QqTOF (double quadrupole-TOF) or Q-orbitrap (quadrupole-orbital ion trap) [65]. While exhibiting high sensitivity, selectivity and mass accuracy, LC-HRMS is a very high-cost technique, and needs recurrent maintenance and to be regularly calibrated to maintain the high mass accuracy and resolution. In addition, its application depends on the training of users and data file storage because, when using HRMS in full scan mode for large numbers of samples, lots of information must be processed and stored [92].

Recently, multi-mycotoxin methods have been developed to determinate a greater number of mycotoxins in a single chromatographic run. This progress is relevant since one food item may be contaminated by a fungus that produces different mycotoxins or can be contaminated by more than one species of fungus, resulting in co-occurrence [63,94]. However, one of the challenges is the matrix effect; the signal is often suppressed due to co-elution with matrix components. Matrix-matched calibration, the addition of standard or use of internal standard are some of the solutions. Matrix-matched calibration uses calibration standards for fortifying “blank” samples (without mycotoxins of interest), with the addition of known mycotoxin concentration, and it is expected that the impact of the matrix effect on the response of mycotoxins is similar in calibration and samples [93]. The internal standard (IS) allows greater flexibility in extraction techniques and conditions since it has previously been added to the sample. Moreover, IS allows correction of signal variations, measuring the relative response ratio between a mycotoxin and IS and associated recovery of method to final result [95]. Some of the most commonly used ISs in AF determination are isotopes, such as ^13^C-aflatoxin, and deuterated aflatoxin, since they will have characteristics similar to AF [92]. However, for correct analyses concerning multi-mycotoxins, a labelled compound for every single mycotoxin of interest should be used. Zearalanone (ZAN) is also an internal standard widely used [16,66,96], with chemical structure and chemical behavior during extraction and analysis similar to mycotoxins, but there is a risk of natural contamination of samples.

In the scientific literature (Table 1), the widely used analytical column is C18 with 150 × 4.6 mm, and particle size of 5 μm. Most recent studies with UHPLC used sub-2 μm diameter particles and permitted the reduction of LC column length to 100 × 2.1 mm [60,77,86] and 50 × 2.1 mm [71,78,97]. Towards the mobile phase, the most used solvents are water, acetonitrile and methanol, with the addition of formic acid, acetic acid or ammonium formate, in different proportions and mixtures. Regarding LOD, the methods just for AFs present lower LODs, as Nonaka et al. [67], with the lower LOD of 0.02 μg/kg, also Reza et al. [70] and Alsharif et al. [76] with 0.05 μm/kg. Concerning multi-mycotoxins methods, the lowest LOD is 0.17 μg/kg from Arroyo–Manzanares et al. [71], and the lowest LOQ is 0.05 μg/kg [75,86] but the minor range of LOQ is in Bessaire et al.’s study (0.05–0.25 μg/kg) [86].

Chromatographic techniques have been commonly used in the determination of aflatoxins and other mycotoxins, with good results, in particular excellent sensitivity and the ability to detect multiple analytes in low levels in complex matrices, but require expensive equipment and trained personnel and high maintenance costs, and may not be a technique accessible to all countries and/or laboratories [35,98]. There is a need to develop faster, cheaper and simpler methods [79] to improve and facilitate the control of mycotoxins in order to ensure food safety.

#### 8.1.2. Immunoassays

Immunochemical methods are emerging as new methods for the determination of mycotoxins, based on the specific and high affinity reaction between the antigen (the target (bio)analyte) and antibody [98]. Enzyme-linked immuno-sorbent assay (ELISA) is one of the immunoassays with antibodies fixed on a solid base, able to distinguish the three-dimensional structure mycotoxins, causing the specific bond [79]. This technique requires antibodies produced by immunizing animals with mycotoxins, including rabbits and goats. However, mycotoxins with low molecular weight do not produce immune responses by themselves. Therefore, mycotoxins are conjugated with a carrier protein or polypeptide before immunization in order to stimulate immunological response and production of antibodies [98]. Conjugation depends on the chemical structure and functional groups of mycotoxins. AFs do not have a reactive group, so a carboxylic group is primarily introduced [98] and later conjugation with bovine serum albumin-BSA [99]. Cross-reactivity of antibodies, that is, the ability of antibodies to react with other antibodies, influences the accuracy of the assay [64]. For example, Leszczynska et al. [100] demonstrated that all antibodies used to determine total aflatoxins tested positive for cross-reactivity (aflatoxin B1 100%, AFB2 200%, AFG1 15%, AFG2 16%, AFM1 63%). Most monoclonal antibodies produced against aflatoxins are highly specific to AFB1 and have a partial cross-reaction with AFG1 [101]. Other compounds with similar chemical groups can also interact with antibodies, due to low molecular weight, resulting in underestimations or overestimates [79].

ELISA has two main steps: (1) Reaction between antibody and antigen and (2) enzymatic reaction between enzyme and substrate. The assay occurs in a well of a test plate, which contains antibodies selective to antigens of interest immobilized in a solid phase. Then, another antibody, conjugated with an enzyme, binds to immobilized antigens. The enzyme substrate is added, and a reaction occurs that involves color change measured and compared with calibration curves, allowing quantification of antigens [93].

**Figure 3:**
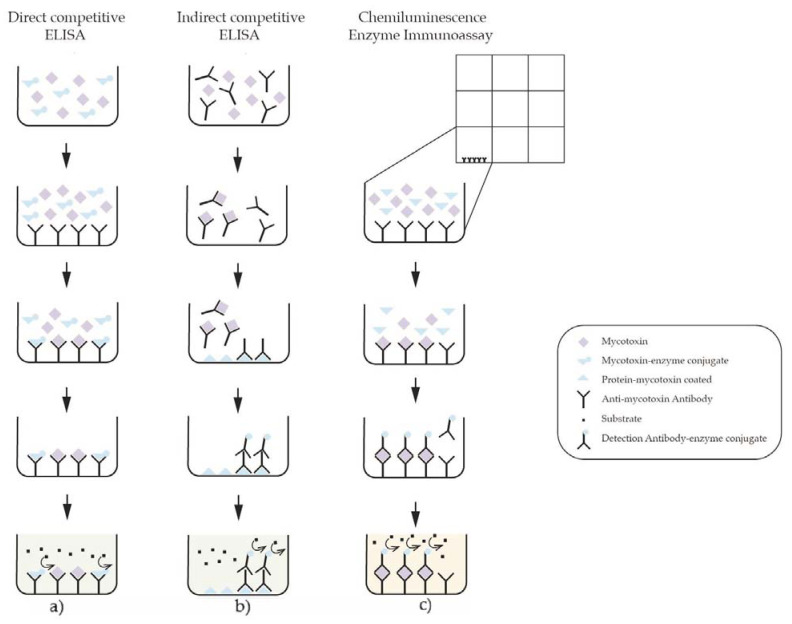
Schematic illustration of Immunoassays: (**a**) Direct competitive ELISA; (**b**) Indirect Competitive ELISA and (**c**) Chemiluminescence Enzyme Immunoassay.

There are variations of this assay, depending on the characteristics of the antigen and matrix. Competitive ELISA assay is based on competition for antibody binding sites [64]. There are two versions of competitive ELISA: Direct and indirect (Figure 3).

Direct ELISA uses a mycotoxin-enzyme conjugate that competes for the available spaces on the coating antibody layer, while indirect ELISA involves a protein–mycotoxin conjugate immobilized on the microplate that competes with mycotoxin present in the sample [64,93]. The most commonly used enzyme is horseradish peroxidase (HRP) and alkaline phosphatase (AP) [35,79]. In direct competitive ELISA, the sample solution or mycotoxin standards are mixed with a mycotoxin coupled enzyme and are added to wells coated with antibody. Thus, there is competition of mycotoxins with mycotoxin conjugated by binding to the antibody. This is followed by a washing step to remove any unbound enzyme conjugate. After that, an enzymatic substrate is added; enzyme converts substrate into a color product. The reaction is interrupted by adding a stop solution and color intensity is measured spectrophotometrically with an absorbance filter of 450 nm [79,93,102]. In indirect competitive ELISA, antibody is added with sample solution containing mycotoxins. Next, the solution is added to wells coated with protein–mycotoxin conjugate, and the remaining free antibodies bind to mycotoxins in wells. After washing, a second antibody labelled with an enzyme detected the first antibody [79,93,102]. Then, the enzymatic substrate is added, and the enzyme converts the substrate into a color product. In these assays, color intensity is inversely proportional to the concentration of mycotoxins in the sample [79]; that is, the higher concentration of mycotoxin, the lower signal generated, since there is less mycotoxin conjugated with the enzyme or less second antibody labelled with an enzyme.

While direct ELISA uses a single conjugate, requires one less incubation step and, consequently, one less washing step [101], indirect ELISA is more sensitive and flexible since more than one second antibody can be bound per primary antibody [93]. On the market, ELISA kits based on the direct competitive assay for the test of aflatoxins in different food matrices are already available, including in nuts. In pistachio nuts, ELISA is used for rapid methods for mycotoxin detection. Lee et al. [103] developed rapid direct competitive ELISA for monitoring aflatoxin AFB1 at 10 μg/kg in pistachio and other nuts and cereals. Bensassi et al. [104] studied the contamination of pistachio nuts in two years of storage, screening levels of AFB1 by ELISA combined with an immunoaffinity step. Some biosensors based on indirect competitive immunoassay for the detection of AFB1 have been developed for different matrices, like cereals [105,106], and peanuts [105,107].

Several studies have compared the determination of mycotoxins by ELISA and HPLC method, since HPLC is considered a reference method and widely used [35]. For example, Azer and Cooper (1991) analyzed 178 food samples for total aflatoxins, including nut and nut products, obtaining a correlation coefficient of 0.999, i.e., there is a high degree of agreement between the two methods. It should also be noted that the ELISA method demonstrated a high degree of precision, useful for rapid testing, in a concentration range of 15 to 50 μg/kg [108]. Moreover, Reza et al. [70] and Ostadrahimi et al. [73] used the ELISA method to determine AFs in pistachio and other nuts, and the results were favorably confirmed by HPLC. Contrary to HPLC, ELISA is not useful in multi-mycotoxin determination because it requires different assays with different antibodies specific to each mycotoxin [103,104] or group of mycotoxins [109], becoming more expensive and more time consuming.

#### 8.1.3. Biosensors

The chromatographic methods are expensive and require trained personnel and the procedures are, in general, complex and slow for multiclass residues. For these reasons, a new technology is necessary to simultaneously detect different compounds including mycotoxins.

In general (bio) sensors provide fast, reliable screening, with good sensitivity and selectivity, and low detection limits and are relatively economic, especially if applied to a large number of routine analyses. The detection of mycotoxins by biosensors mostly relies in two types of detection methods: Optical and electrochemical [15]. The current trend is the optical biosensors based on chemiluminescent methods, which can be divided into CLIA (chemiluminescent immunoassay) and CLEIA (chemiluminescent enzyme immunoassay) (Figure 3).

CLIA detection is the result of a very selective (bio)chemical reaction between the antigen (the target (bio)analyte) and an antibody specific to detection of the target (bio)analyte. The reaction mechanism is based on oxidation and reduction reactions that yield changes in chemiluminescence, depending on the amount of target analyte that can be monitored by optical detection methods. The most commonly utilized chemiluminescent (CL) compound in aqueous solution is luminol or isoluminol. In the presence of a catalyst (enzyme, metal-containing molecule or metal), luminol interacts with hydrogen peroxide in alkaline solution to produce 3-aminophthalate in an excited electronic state, which returns to the ground state with the emission of light. The signal is then detected by an optical detection system. To increase the lifetime and the amplitude of the signal, a substance known as an “enhancer” (for example, 4-iodophenol) is added to the reaction medium. At the end of an immunoenzymatic experiment, this luminous reaction can be used to detect antigen–antibody binding [110].

CLEIA (combines chemiluminescence (CL) and enzyme immunoassay) detection techniques are currently the most sensitive in immunoassay research. CLEIA is becoming increasingly popular for the detection of trace compounds due to its great qualities of high specificity, lower limit of detection, good linearity range and environmental friendliness [111]. The main two label enzymes used in CLEIA are horseradish peroxidase (HRP) and alkaline phosphatase (ALP). Due to the low cost and the ease of access, horseradish peroxidase is considered the most used. While the luminescence efficiency of the horseradish peroxidase system can be increased by using a suitable enhancer [111], it is quite poor when compared to the ALP system [111]. In any case, CL substrates, such as the luminol/peroxide/enhancer system for horseradish peroxidase (HRP) or dioxetane-based substrates for alkaline phosphatase, can efficiently detect enzyme labels.

One of the advantages of the CLEIAS is the possibility of application of advanced nanotechnology. For example, Freitas, Barros, Brites, Barbosa and Silva (2019) used the Evidence Investigator Biochip Array Technology (BAT) (Randox, Crumlin, UK) in a semi-quantitative methodology in the analysis of mycotoxins in maize [112]. In this case, Biochips were used, composed of 9 mm square-shaped solid substrate with a panel of discrete test regions (DTR) where each DTR consists of different antibodies or other reactive species specific (multiplexing) to each assay. The advantage of being able to detect and semi-quantify, in a single analysis, multiple analytes, makes CLEIA a powerful screening tool in several matrices.

While (bio)sensors are a trend and numerous have been developed during the last years, there is a lack of application of this methodology to determine mycotoxins, especially in pistachios. Kumaniaris et al. [113] developed an electrochemical immunosensor for the determination of AFB1 in pistachio based on the immobilization of the AFB1 antibodies on the surface of gold screen printed electrodes. This method presented good sensitivity (LOD = 1 ng/mL) showing potential as a screening method, but also as a quantitative method since it successfully determines AFB1 concentrations in the range of 4.56–50.86 ng/mL in unknown pistachio samples.

Spectroscopy techniques have been applied for rapid and real-time analysis for mycotoxins, with little or no sample preparation, without destroying the sample [114]. Paghaleh et al. [115] developed a method based on the laser induced fluorescence spectroscopy, using a UV laser (λ = 308 nm) for in line measurement of the concentration of AFs in pistachio nuts, without sample preparation, and results are in agreement with the HPLC method. Wu and Xu [116] developed a multiplexing fiber optic laser induced fluorescence spectroscopy for detection of AFB1 in pistachios, using five wavelengths between 440 and 564 nm because physical and chemical characteristics of pistachios at different positions of contaminated products are unequal or nonuniform. Results show an accuracy of 97% and low levels of AFB1 (50 ppb). Valasi et al. [117] used diffuse reflectance infrared Fourier transform spectroscopy with chemometrics for screening AFs in pistachios using four spectral regions to classify AF-contaminated from non-contaminated pistachios and results show that this methodology correctly separated 80% of test samples.

## 9. Occurrence of Mycotoxins in Pistachios

In pistachio nuts, AFs are the most frequently found mycotoxins (Table 2). The occurrence of AF contamination is sporadic and very dependent on environmental conditions [11]. In nuts, FAO indicates that *Aspergillus flavus* and *A. parasiticus* do not grow or produce aflatoxins at temperatures below 10 °C, relative humidity below 70% and water activities (aw) lower than 0.7 [118]. According to Baazeem et al. [119], *A. flavus* grows in pistachio when incubated at between 25 and 35 °C and with aw ranging from 0.95 to 0.98, in in vitro and in situ studies, but AFB1 was optimally produced at 30 °C and aw >0.98. These mycotoxins are predominant in Africa, Asia and North and South America, where environmental conditions are more favorable. However, due to globalization and climate change, AFs can be found all over the world [120]. Beside nuts, AFs occur in various other foods, namely cereals (corn, rice, wheat), spices (pepper, turmeric, ginger), oilseeds (peanuts, soybeans, sunflower) and legumes, among others [39].

AFs were first identified in England, in the 1960s, where an outbreak arose, known as “Turkey X disease”, which caused the death of more than 100,000 turkeys due to consumption of peanut flour contaminated by fungi, namely species such as *Aspergillus flavus* and aflatoxins [13]. The first outbreak of aflatoxicosis in humans occurred in 1974 in India and caused 106 deaths due to consumption of contaminated maize from environmental causes that occurred before harvest [32]. In Kenya, in 2004, one of the largest and most severe outbreaks of aflatoxicosis occurred in humans, which caused the death of 125 people due to liver failures due to consumption of contaminated maize, with more than 300 cases of abdominal pain, pulmonary edema and liver necrosis [124]. This outbreak was due to incorrect storage of maize in a humid and hot environment, providing for the growth of fungi, combined with a poor diet among the low socio-economic population and also a lack of medical resources [125].

The vast majority of studies summarized in Table 2 present high values of positive samples; however, sampling is reduced and may not be representative of the global market. Cheraghali et al.’s study [24] comprises a greater number of samples, collected between March 2002 and February 2003 in Iran; 37% of samples were contaminated with AFB1 and 11.8% were above maximum levels in the country (5 μg/kg), higher than that legislated in Portugal and Europe. About 28% of the samples were contaminated with all AFs. AFB1 is the most frequently found and most concentrated. In some samples, the maximum levels were exceeded, constituting a risk to the health of the population, particularly in the study by Diella et al. [74], which presented the highest levels of AFB1 and sum of AFs, El Tawila et al. [72], Ali Alsharif et al. [76] and Cheraghali et al. [24]. El Tawila et al. [72] showed that AFB1 content in pistachio nuts has the highest amplitude, ranging from 1.9 to 411 μg/kg, and in the study by Diella et al. [74] values of AFs are between 8.8 and 387.3 μg/kg.

In Europe, the occurrence data on food as submitted to EFSA, resulting from samples collected between 2003 and 2018 to reflect the current contamination levels in European countries, show that the food category “Legumes, nuts and oilseeds” is one of the greatest contributors to dietary exposure to AFs and AFB1, and the highest AF mean concentrations are in pistachios, peanuts and other seeds [37]. Previously, pistachios also had the highest level of AFs compared with other tree nuts [23]. In Iran, the main producing country, the mean concentrations of AFT in pistachio was 54 μg/kg and considering the maximum level of 4 μg/kg and 20 μg/kg, 40 and 60% of pistachio samples were rejected, respectively [126]. JECFA conclude that pistachios were the main contributor to dietary AF exposure from tree nuts, ranging from 0.2 to 0.8 ng/kg bw per day [127]

Few studies have evaluated AFs in nuts and derivatives with different types of processing. Ostadrahimi et al. [73] determined AFs in raw pistachios and roasted with salt, demonstrating that samples toasted with salt contained higher AFs than unprocessed samples, in the order of 22.02 μg/kg. This fact may be due to prolonged storage time with conditions suitable for fungal growth in addition to thermoresistance and stability of AFs at processing temperatures. AF occurrences were sometimes different from study to study depending on the characteristics of the samples analyzed. Some studies have not detected AFs in the food matrices [67], but other studies reported high levels of contamination. This is justified by the different origins of products (not mentioned in many cases), different storage conditions or type of processing.

Several studies evaluated the OTA levels in pistachio nuts [25,60,76,122,124,128] and the results show low percentage of positive samples for OTA contamination. In the study by Liao et al. [60], three of the pistachio samples were contaminated with OTA, between 1.0 and 6.6 μg/kg. Moreover, Varga et al. [25] and Zinedine et al. [128] noticed the presence of OTA, but at levels lower than LOQ.

Fernane et al. (2010) conclude that pistachio can be highly contaminated with aflatoxin- or ochratoxin-producing isolates but the presence of mycotoxins is not high. In fact, out of 31 samples, only two samples were contaminated with AFs and only one sample had OTA [68].

None of the studies in this review mentioned the presence of other mycotoxins, such as DON, FB1, FB2, ZEA, T2 or HT2, in pistachios. In summary, the available data is still scarce in pistachio nuts and the evaluation of the effect of processing is lacking.

Regarding emerging mycotoxins, few studies have evaluated the presence of these mycotoxins in pistachios. Tolosa et al. [129] surveyed the occurrence of ENNs and BEA in nuts and dried fruits in Spain, studying three samples of pistachio. Results show that no presence of BEA and ENNs in pistachio fruit is detected, but, in pistachio shell, ENA, ENA1 and ENB are found at concentrations of 0.326 μg/kg, 0.015 μg/kg and 0.209 μg/kg, respectively, explained by the protective effect of the shell. FUS is produced by *Fusarium proliferatum*, *F. subglutinans* and *F. verticillioides* and occurs in grains and grain-based foodstuff [55]. STC, an *Aspergillus* mycotoxin, mainly occurs in grain, green coffee beans, spices, nuts and cheese, but information is still limited. Concerning *Alternaria* mycotoxins, TeA is the most frequently found in nuts like almonds, hazelnuts, peanuts and pistachio [68] and are probably associated with negative effects on protein biosynthesis [60].

## 10. Biomonitoring

Biomonitoring is an important method for assessing the real exposure to aflatoxins by humans, determining concentrations of mycotoxins, their metabolites or reaction products in biological fluids [130]. It involves the collection of biological samples from individuals, such as blood, urine, saliva, breast milk, as well as hair and nails. To do this, it is necessary to have knowledge of toxicokinetics, especially biotransformation, to identify possible biomarkers of exposure. Biomonitoring is currently an area under development; determination of AFs in food does not constitute a true assessment of exposure since individuals are exposed to multiple food sources with aflatoxins, in addition to other routes of exposure, such as inalatory and dermal [47].

In the case of AFs, biomonitoring can be performed by analyzing the presence of AFB1 metabolites in blood, milk and urine. In addition, excreted DNA and protein adducts in blood can also be monitored [14]. AF metabolite evaluation in biological fluids is usually performed through liquid chromatography coupled with tandem mass spectrometry (LC-MS/MS). Based on studies in humans and animals, adduct AFB1-N7-guanine in urine represents the most reliable biomarker for exposure to aflatoxin, but reflects only recent exposure. In addition, AFB1-albumin adduct is also considered a biomarker for prolonged blood exposure to AFB1 due to the half-life time of albumin of 20 days. AFM1 can be found in human breast milk, which can be considered as a biomarker of maternal and infant exposure to AFB1. It is also excreted in urine and can be considered a biomarker, however, only for recent exposure to aflatoxins [14,47].

In Portugal, Martins et al. [130] evaluate the exposure of the population to mycotoxins, between 2015 and 2016, through analysis of 37 biomarkers in 24 h urine samples and first morning urine, to estimate probable daily intake and perform risk characteristics. From this study, it was concluded that the Portuguese population is more exposed to six mycotoxins: Deoxynivalenol, zearalenone, ochratoxin A, alternariol, fumonisin B1 and citrinin. The levels are above safety limits, representing a public health problem. In this study, exposure to aflatoxins was not evaluated.

## 11. Prevention and Control

Due to the risks of aflatoxins to human health and economic losses, strategies have been developed to reduce *Aspergillus* and AFs contamination. Prevention of fungal contamination is the most effective and preferable measure.

Reducing levels of AFs in pre-harvest begins with plant selection, planting and harvesting dates, plant density and crop rotation, as well as soil treatments, irrigation and pest management [49]. AF contamination can be prevented using seeds genetically modified to be resistant to *Aspergillus* infection and/or environmental stress [34]. However, plant breeding could not be effective because resistance is conferred by multiple genes and environmental pressure is an uncontrollable factor [131].

Another biological pre-harvest strategy to reduce AFs is using non-toxigenic/atoxigenic *A. flavus* isolates to competitively exclude aflatoxin-producing strains during crop colonization or physical displacement. To ensure efficacy, atoxigenic fungi must be (1) selected from local environments, (2) highly competitive and (3) predominant relative to the toxigenic strain in agricultural environments. This strategy shows a reduction in AF contamination of between 70 and 90% in cotton, maize and peanuts, and it has also been implemented in pistachios with reductions ranging from 20 to 45% [131,132,133]. Several of the atoxigenic *A. flavus* strains have been developed into biopesticides for the management of AF contamination and they are already used in the USA in pistachios, such as AF36 coated into a carried sterile grain [134]. However, there are uncertainties regarding their use, mainly (1) the impact of the addition of biocontrol strains in *Aspergillus* population, like a decrease of *A. flavus* followed by an increase in *A. niger* [135] and (2) the possibility of atoxigenic strains reverting back to toxin producers [136]. Some *Aspergillus* strains could be also effective in post-harvest AF mitigation [132].

While the pre-harvest contamination with AFs is more common in tree nuts than post-harvest contamination [131], in post-harvest, control of moistures, temperature, mechanical damage, insect damage and aeration can prevent mycotoxin contamination.

Another prevention strategy is predictive modelling, using large volumes of data and various correlate environmental factors with the potential for *A. flavus* growth and consequently aflatoxin production in the entire food chain [136]. In pistachio storage, Marín et al. [137] applied models to predict the growth of *A. flavus* and AF production as functions of moisture and temperature and results show that the model correctly predicts the presence of *A. flavus* in 90% of cases and AFs in 89% of cases. Aldars-García et al. [138] also attempted to model growth and AFB1 production by *A. flavus* in storage and transport in order to support decisions on ventilation timing and refrigeration adequation, respectively; the model correctly predicted the presence of AFB1 in 70 to 81% of cases. While post-harvest modelling is more developed, preventing contamination in pre-harvest is also a good perspective. Kaminiaris et al. [139] developed a mechanistic model considering a tree’s phenology and meteorological data which correctly predicted 75% of AFB1 contamination in pre-harvest; the authors suggested that this model could indicate the appropriate time for harvest, supporting agricultural systems, and also the pistachios with the highest risk of contamination due to the prediction of field conditions. Predictive modelling has also been applied to OTA in pistachio by Marín et al. [140], who built a probability model function of moisture and temperature, correctly predicting 90% of the cases.

Recently, metabolomics was applied to future prevention of mycotoxins. This new science analyzes metabolomes, all the low molecular weight metabolites in biological samples, as a result not only of the cell’s genome but also of the environment interaction. Metabolomics is useful for understanding the chemical interactions between plant, toxigenic fungus and microbiota, and the influence of biotic and abiotic stress in biosynthesis of mycotoxins and modified forms of mycotoxins as a result of biological or chemical modifications, such as food processing. The knowledge of determinants and factors that govern fungus infection and mycotoxin production allows the development of new efficient strategies to mitigate the occurrence of mycotoxin in food [141].

## 12. Decontamination

In order to reduce or eliminate AF contamination and to ensure food safety, decontamination methods can be physical, chemical or biological. The effectiveness depends on several factors, such as the chemical stability of mycotoxins, the nature of the process, the type and the interaction with the food matrix and the interaction with multiple mycotoxins [142]. It should always be taken into account that these methods should: (1) Inactivate, destroy or remove the toxin; (2) not be able to produce or leave toxic residues; (3) necessarily maintain the nutritional value of the food; (4) not change the acceptability or technological properties of the food; and (5), if possible, destroy fungal spores, preventing the proliferation and production of new mycotoxins [142]. Table 3 presents the outcomes from studies on aflatoxin B1 decontamination by physical, chemical and biological methods.

### 12.1. Physical Decontamination

Physical processes of decontamination include separation of the density-contaminated fraction and the reduction/inactivation of AFs by cooking, boiling, toasting, microwave heating or irradiating contaminated food. However, AFs are highly heat stable and are not easily destroyed, so it is necessary to heat at high temperatures to effectively decrease the levels of aflatoxins [34], depending on time, temperature and moisture content [13]. Some studies indicate that roasting aflatoxin-contaminated pistachios at 150 °C for 30 min reduced AFB1 levels by 63%; when the same process was performed, for 120 min, more than 95% of the AFB1 was degraded, but changes were caused in the appearance and taste of the pistachios [12,143]. Ostadrahimi et al. (2014) determined AFs in raw and roasted with salt pistachios, demonstrating that the samples roasted with salt contained higher content of AFs (mean: 22.02 μg/kg) than the unprocessed samples (mean: 0.48 μg/kg) [73]. This fact, according to the authors of the study, may be due to the prolonged storage time in conditions suitable for fungal growth. According to Yazdanpanah et al. (2005), the effect of toasting on the reduction of AFs in pistachios was evaluated to define the optimum conditions [143]. It was found that the treatment of samples at 150 °C for 30 min significantly reduced the levels of AFs, without alteration of organoleptic characteristics. Heat treatment is widely applied in the food industry, for biscuits, pasta, cereals, snacks, etc. [154].

Recently, non-thermal processes like Cold Plasma treatment, electron beam irradiation and pulsed electric field have been applied to reduce mycotoxin contamination with good results in different foodstuffs [155,156,157,158]. These techniques are processed at near room temperature, and so do not significantly affect the nutritional status or the organoleptic properties, constituting alternatives to the conventional decontamination techniques for pistachio [159,160,161]. Cold plasma treatment (CP) is an interesting tool to reduce mycotoxins due to both fungi reduction and mycotoxin degradation in all food chains [162,163]. CP was already applied by Tasouji et al. [164] in pistachio nuts to reduce *Aspergillus flavus* and results showed a reduction of 67% with 10 min of irradiation time, without alteration of the texture. The CP technique was also performed to reduce AFB1 in hazelnuts and reduced 70–73% of spiked AFB1 [165]. This decontamination process is possible for industrial implementation because it is eco-friendly, energy efficient, low cost and fast. Gamma (γ) and ultraviolet (UV) radiation are also applied for destroying AFS because they are photosensitive [13]. Ghanem et al. [144] studied the effect of gamma radiation on the inactivation of AFB1 and concluded that at a dose of 10 kGy there was a reduction of 68.8% and 84.6% in shelled and in-shell pistachios, respectively, and degradation was positively correlated with the increase of dose. There is a significant difference between shelled and in-shell pistachios; the authors explain this due to the fact that in in-shell pistachios the fungal growth was limited to the surface of the peel and limited *Aspergillus* entrance into the kernel itself. These techniques are applicable to different food matrices. However, due to the associated risks to human health, more studies are needed [34].

Currently, mechanical separation based on size and density by gravity systems removes small and shriveled pistachios. Additionally, manual sorting of stained shells, discolored shells and defective pistachios is also applied in industry. Both methods are applied to reduce AF contamination in pistachios [154,166,167,168]. Several studies indicate a positive correlation between physical properties (size, color, shape, density and fungal damages) and AF contamination. Doster and Michailides [169] reported that pistachio nuts with oily shells, crinkled shells or shell discoloration had more kernel decay and NOW infestation, and consequently more AF contamination. Shakerardekani et al. [170] concluded that pistachios with yellowish-brown and dark-greyish stains have the highest levels of AFs, and, after removing those stained nuts, there is a contamination reduction of between 95 and 99%, depending on pistachio cultivar. Manual sorting is a more time-consuming and tedious task, so sorting using new technologies has been studied. McClure and Farsaie [171] reported the elimination of pistachios contaminated with AFs by fluorescence sorting. Özlüoymak and Güzel [172] develop an image processing technique to measure and analyze color by irradiating pistachios at a wavelength of 365 nm and the contaminated pistachios exhibited bright-greenish yellow fluorescence. This method can be applied at a new real-time determination and separation system.

Furthermore, some pistachios could look healthy on the outside but have necrotic spots resulting from stigmatomycosis disease, which has a positive correlation with higher aflatoxin contamination. In addition, Yanniotis et al. [173] developed a method based on X-ray imaging for the detection of necrotic spots in pistachios and rejecting these nuts results in a reduction of AFs of 60%. This methodology could be applied in an automatic separation machine at industrial levels to reduce AF contamination.

### 12.2. Chemical Decontamination

Chemical decontamination methods use chemical compounds that degrade the structure of AFs. These methods may result in toxic degradation products that may harm the consumer’s health, and/or unacceptable changes in the quality of the final product, both nutritional and sensory [11]. Within the chemical methods, three are highlighted: Acidification, ammonization and ozonation.

Acidification is a way to prevent fungal growth or inactivate AFs. Lactic acid, citric acid, tartaric acid or hydrochloric acid are used more frequently and the use of salicylic, benzoic, boric, oxalic or propionic acids has been shown to be effective in reducing the content of AFs. In the case of AFB1, the use of acids results in the conversion to AFB2, AFB2a, AFD1 and less toxic forms [13,29,31,154]. This is a simple method, without the need for equipment or specialized people, and only requires the contact of the food matrix with acid for a certain period of time [34], and is a low-cost technique.

Ammonization is the most efficient technique, with a reduction of about 99%, but more common in animal feed decontamination. Ammonium is used in gaseous or hydroxide form, degrading AFB1 in AFD1 in alkaline, and consequently reducing mutagenicity. This technique requires more complex infrastructure [34].

Ozonation is one of the most promising methods, using gaseous ozone, a potent oxidant, for short periods of time. It is very effective in different types of food matrices and is accepted to be used at the industrial level [34,154]. Akbas and Ozdemir (2006) study the efficiency of ozone in the degradation of AFs in pistachios, and the results indicated that ozonation at 0.9 mg/L for 420 min reduced AFB1 and AFs by 23% and 24%, respectively, which indicated that AFB1 is more sensitive to this method than the other aflatoxins (AFB2, AFG1 and AFG2). In addition, no significant changes occur in color, fatty acid composition or organoleptic properties of pistachios [145].

Another method for decontamination is the association of two or more types of processes. Rastegar et al. (2017) use physical and chemical methods through roasting 50 g of pistachio nuts at 120 °C for 1 h with 15 mL lemon juice and/or 6 g of citric acid to remove AFB1. The level of AFB1 was reduced by 49% without a noticeable change in the desired appearance of pistachios. The reduction was higher (93%) using 30 mL lemon juice but desired physical properties were altered [153].

It is also worth mentioning the use of aqueous extracts of plants, since they are rich in bioactive compounds such as tanines, terpenoids, alkaloids and flavonoids, with antifungal properties [34,154]. Several studies have indicated the high efficiency in the degradation of AFs with the use of plant extracts. The authors indicate that detoxification is related to the modification of the lactone ring structure of the AFs [148,151]. The extracts will have high molecular weight compounds, be soluble in water and be thermolabile [146,147]. In the case of *A. vasica* extract, alkaloides appear as a principle of detoxification of aflatoxins [147]. All studies reveal a reduction in the contents of other aflatoxins, especially AFB2 [146,147,149]. While this technique is more time consuming, it is simple since the sample is incubated with plant extract in specific time and temperature conditions, it has high efficacy and, because it is considered “natural”, it is more acceptable to the consumer. These extracts have compounds that are biodegradable, environmentally friendly, safe and low cost, constituting an alternative to other synthetic chemical compounds [154]. However, the standardization of the extract activity must be assured, since the composition of the extract is influenced by edapho-climatic conditions, and different cultivars. A drawback is the influence on the organoleptics characteristics of the foodstuffs that can be overcome by micro- or nanoencapsulation [147,174,175].

### 12.3. Biological Decontamination

Biological methods use bacteria, yeasts or enzymes to degrade or inactivate AFs, or, in some cases, for adsorption of mycotoxins. Lactic acid bacteria, such as *Lactobacillus*, and *Saccharomyces cerevisiae*, are among the most studied for this process, especially in fermented products and beverages. The food is inoculated with the microorganism, so this method is more complex and time consuming, because it requires the growth of the microorganism. The enzyme peroxidase decomposes hydroperoxides and free radicals are generated that react with aflatoxins [29]. For example, kefir-grains are a symbiotic association of microorganisms and Ansari et al.’s (2015) study indicated a 96.8% reduction of AFG1 in pistachio with kefir grains pre-treated at 70 °C and incubated for 6 h at 30 °C [150]. *Bacillus subtilis* UTBSP1 have the ability to reduce AFB1 by 95% as shown in Farzaneh et al.’s (2012) study, resulting from incubation at 35–40 °C for five days, and its degradation activity was likely due to the extracellular enzymes [151]. Yeasts are also studied for decontamination, for example, *Saccharomyces cerevisiae* has the ability to surface binding aflatoxin in 40% and 70%, depending on the initial AF concentrations (10 ppb and 20 ppb, respectively) and the study also showed that acid and heat treatments increase this ability to 60–73% and 55–75%, respectively. This treatment had no effect on qualitative characteristics of pistachio nuts, such as color and texture [152].

## 13. Conclusions

Food fungal contamination is a hot topic receiving considerable attention by researchers and the food industry. While it is important to understand the mechanisms and conditions that favor the production of mycotoxins by fungi in foods, it is also important to monitor the levels of contamination of highly consumed food products such as nuts, to ensure consumers’ health. Pistachio is considered a “healthy food”, due to its nutritional level and its health benefits, so research is of utmost importance to predict the reduction of mycotoxin contamination. The development of analytical methods, screening and confirmation is of utmost importance in nuts, where the extraction procedures are very complex, mainly because of the high fat content. Most published studies agree that QuEChERS extraction followed by HPLC-MS/MS for detection/quantification is the most suitable analytical method to carry out multi-mycotoxin determination in pistachios allowing achieving low detection levels with higher sensitivity, selectivity and specificity. In fact, multi-target methods are the most useful due to the co-occurrence of mycotoxins in food. The detection of multiple mycotoxins employing HRMS hybrid detectors as QqTOF or QOrbitrap will have the most impact in the near future, due to their potential for high-throughput analysis and more accurate mass measurement. Moreover, new methods should be targeted to research emerging mycotoxins due to their negative effects on human health and lack of information available concerning the occurrence in nuts. However, the previous use of screening methods is of great importance due to their simplicity and rapid throughput.

The information presented here suggests that the number of monitored mycotoxins in pistachios increases with a vast range of physicochemical properties and proper research on the means of decontamination should be conducted to reduce the number of nuts that are rejected due to mycotoxin contamination. Heat treatment is widely applied in the food industry but the use of plant extracts has aroused more interest because it is more “natural” and has fewer health effects. However, there are difficulties related to standardization: Availability varies throughout the year and there is variability in composition among, for example, different cultivars and diverse edaphoclimatic conditions.

## Figures and Tables

**Table 1:** Summary of analytical methodologies used for mycotoxins determination in pistachio.

Type of Sample	Analytes	Clean-Up Methods	Procedure of Extraction	Detector	Conditions	Analytical Column	Internal Standard	LOD (μg/kg)	LOQ (μg/kg)	Reference
Pistachio	AFB1; AFB2; AFG1; AFG2	IAC	Sample quantity: 125 g Sample extraction: 475 mL MeOH/H_2_O/Hexane (63:16:21 *v/v/v*); filtration, dilution with water; IAC:10 mL PBS; 75 mL filtrate; wash 15 mL H_2_O, vacuum; elution with 0.5 mL MeOH	HPLC—FLD with PCD	Mobile phase: H_2_O/MeOH/ACN (42:29:17, *v/v/v*) Flow-rate: 1 mL/min Temperature column: Injection volume: 100 μL λexcitation: 365 nm λemission:450 mm	C18 250 mm × 4.6 mm 5 μm	-	0.1–0.4	-	[24]
Peanuts, pistachio, wheat, maize, cornflakes, raisins and figs	AFB1, AFB2, AFG1, AFG2, OTA, DON, FB1, FB2, T2, HT2, ZEA, CIT, etc	-	Sample quantity: 25 g Sample extration: 100 mL ACN/H_2_O (80:20 *v/v*), shaken 2 h, diluted 1 mL extract with 3 mL H_2_O, filtration	LC-MS/MS	Mobile phase:(A) H_2_O with 0.1% FA (B) ACN with 0.1%FA Gradient program: 90% A at 0 min, 30% A at 12 min, 10% A at 17.5 min, 90% at 21 min (*t* = 25 min) Flow-rate: 0.3 mL/min Ionization: ESI source in the positive mode Temperature column: 30 °C Injection volume: 20 μL Ionization: ESI source in the positive mode Capillary voltage: 2.5 kV Collision gas pressure: 0.8 bar Vaporizer temperature: 450 °C Sheath gas pressure: Auxiliary gas flow: 600 L/h	Alltima C18 150 mm × 3.2 mm 5 μm	-	0.5–200	1–200	[66]
Dried fruits (peanuts, walnut, cashews, pistachio, almond, pecan walnut), cereals, dehydrated fruits and spices	AFB1, AFB2, AFG1, AFG2	SPME	Sample quantity: 0.5 g Sample extraction: 1 mL MeOH:H_2_O (80:20 *v/v*), centrifugation, filtration of supernatant and added to in-tube SPME	HPLC—MS	Mobile phase: MeOH/ACN (60:40, *v/v*):5 mM ammonium formate (45:55) Gradient program: After 8 min, washed with MeOH/ACN (60/40, *v/v*) for 2 min and returned to the initial conditions in 2 min Flow-rate: 1 mL/min Temperature column: 40 °C Injection volume: 10 μL Ionization: ESI source in the positive mode Capillary voltage: 2.5 kV Collision gas pressure: Vaporizer temperature: 350 °C Sheath gas pressure: 30 psi Auxiliary gas flow: 13 L/min	Zorbax Eclipse XD8-C8 150 mm × 4.6 mm 5 μm	AFM1	0.02	0.05	[67]
Pistachios	AFB1, AFB2, AFG1, AFG2, OTA	IAC	Sample quantity: 5 g for AFs and 10 g for OTA Sample extraction: 30 mL ACN/H_2_O (60:40 *v/v*), belnded 10 min, 2 mL extract diluted with 48 mL PBS; Easi-extart AF IAC for AFs and Ochraprep IAC for OTA	HPLC—FLD with PCD	Mobile phase: ACN/H_2_O/acetic acid (51:47:2, *v/v/v*) Flow-rate: 1 mL/min Temperature column: 40 °C Injection volume: 100 μL λexcitation: 333 nm λemission:443 mm	Spherisorb ODS2 150 mm × 4.6 mm 5 μm	-	0.2	-	[68]
Pistachios, walnuts, cashews, almonds, peanuts, seeds, etc.	AFB1	IAC	Sample quantity: 50 g Sample extraction:100 mL H_2_O + 4 g NaCl, 150 mL MeOH, filtration, 5 mL filtrate + 25 mL PBS; IAC:10 mL PBS, 30 mL filtrate, wash 15 mL H_2_O, elution 0.5 mL MeOH, 1 mL H_2_O; filtration if solution not clear.	HPLC—FLD with PCD	Mobile phase: H_2_O/MeOH/ACN (42:29:17, *v/v/v*) Flow-rate: 1 mL/min Temperature column: 40 °C Injection volume: λexcitation: 362 nm λemission:456 mm	Luna C18 25 cm × 4.6 mm, 5 μm	-	0.2	0.6	[69]
Almonds, walnuts, sunflower seeds, sesame seeds, peanuts, pistachios, hazelnuts and cashews	AFB1, AFB2, AFG1, AFG2, AFM1, AFM2	IAC	Sample quantity: 10 g Sample extraction: 33% MeOH, filtration, 500 μL filtrate + 500 μL 33% MeOH	ELISA	Euroclon kit Absorbance at 450 nm	-	-	-	-	[70]
			Sample quantity: 10 g Sample extraction: 1 g NaCl + 40 mL MeOH/H_2_O (80:20 *v/v*) + 20 mL n-hexane, blended for 3 min, eliminate n-hexane phase, filtration; 7 mL filtrate + 43 mL PBS; IAC: 10 mL PBS, 50 mL filtrate, wash 20 mL H_2_O, dried with air, elution 2 mL MeOH	HPLC- FLD with PCD	Mobile phase:ACN/MeOH/H_2_O (17:29:54, *v/v/v*) Flow-rate: 1 mL/min Temperature column: Injection volume: 20 mL λexcitation: 365 nm λemission:435 nm	Hichrom ODS 250 mm × 4.6 mm 5 mm	-	0.05–0.42	0.19–1.4	
Dried fruits (peanuts, almonds, walnuts, pistachios, hazelnuts) and seeds (sunflower, pumpkin, pine nuts)	AFBI, AFB2 AFG1, AFG2, OTA, FB1, FB2, T-2, HT-2, STE, CIT, DON, ZEN	QuEChERS	Sample quantity: 2 g Sample extration: 8 mL H_2_O + 10 mL ACN: 5% FA; 4 g MgSO4 + 1 g NaCl + 1 g sodium citrate + 0.5 g disodium hydrogen citrate sesquihydrate, centrifugation; DLLME for AFs: 2 mL supernatant: evaporation and 1 mL MeOH/H_2_O (50:50), 4 mL H_2_O, 0.21 g NaCl; injection 950 μL ACN + 620 μL chloroform	UHPLC—MS/MS	Mobile phase:(A) H_2_O with 0.3% FA and 5 mM ammonium formate, (B) MeOH with 0.3% FA and 5 mM ammonium formate Gradient program: 0 min:5% B; 1 min:50% B; 2 min:72% B; 4 min:80% B; and 6 min:90% B, finally back to 5 B in 0.2 min and maintained for 1.8 min for column equilibration Flow-rate: 0.4 mL/min Temperature column: 35 °C Injection volume: 5 μL Ionization: ESI source in the positive mode Capillary voltage: 5 kV Collision gas pressure: 30 psi Vaporizer temperature: 500 °C Sheath gas pressure: 50 psi Auxiliary gas flow:	Zorbax Eclipse Plus RRHD 50 mm × 2.1 mm 1.8 um	-	0.17–9.68	0.57–32.6	[71]
Walnuts, pistachios, hazelnuts, cashews, almonds	AFB1, AFB2, AFG1, AFG2	IAC	Sample quantity: 25 g Sample extraction: 5 g NaCl + 125 mL MeOH/H_2_O (60:40 *v/v*), blended for 1min, filtration; 20 mL filtrate + 20 mL H_2_O; IAC: 10 mL filtrate diluted, wash 10 mL H_2_O, elution 1 mL MeOH	HPLC—FLD with PCD	Mobile phase: H_2_O/ACN/MeOH (6:3:1, *v/v/v*) Flow-rate: 1 mL/min Temperature column: Injection volume: 20 μL λexcitation: 360 nm λemission:440 mm	Spherisorb ODS C18 150 mm × 4.5 mm 5 μm	-	0.273–0.536	0.9–1.8	[72]
Cereals and nuts (almond, peanut, pistachio)	AFBI, AFB2 AFG1, AFG2, OTA, OTB, T-2, HT-2, STE, CIT, DON, ZEN, etc.	-	Sample quantity: 1 g Sample extraction: 5 mL ACN/H_2_O (85:15 *v/v*), shaking for 30 min in higher speed with pulsation, centrifugation, 500 μL extract + 20 μL ISs + 480 μL 20 mM ammonium formate, vortex and filtration.	UHPLC—MS	Mobile phase:(A) H_2_O with 0.3% FAand 5 mM ammonium formate, (B) MeOH with 0.3% FA and 5mM ammonium formate Gradient program: 100% A at 0 min, increase to 100% B at 8 min, until 12 min, then, return to 100% A in 8.5 min, equilibration for 5.5 min (*t* = 18 min) Flow-rate: 0.3 mL/min Temperature column: 35 °C Injection volume: 5 μL Ionization: ESI source in the positive mode Capillary voltage: 4 kV Collision gas pressure: Vaporizer temperature: 350 °C Sheath gas pressure: Auxiliary gas flow: 15 L/min	Hypersil GOLD aQ 100 × 2,1 mm -	Isotope labeled ^13^C	-	-	[60]
Pistachios, peanuts and walnuts (raw and roasted with salt)	AFB1, AFB2, AFG1, AFG2	-	Sample quantity: 10 g Sample extraction: 50 mL 33% MeOH, filtration, dilution 1:2 with 33% MeOH	ELISA	Clone total AF ELISA test kit, Absorbance at 450 nm	-	-	-	-	[73]
Almond, hazelnuts, peanuts, pistachio, walnuts, brazil nuts, chestnuts and apricot	AFB1, AFB2, AFG1, AFG2	IAC	Sample quantity: 25 g Sample extraction: 5 g NaCl + 125 mL MeOH/H_2_O (60:40 *v/v*), blended with hight speed 1 min, sediment, filtration of supernatant; 20 mL filtrate + 20 mL PBS; IAC:20 mL diluted filtrate, wash MeOH/H_2_O (25:75 *v/v*), elution 2 mL MeOH + 3 mL H_2_O	HPLC—FLD with PCD	Mobile phase:H_2_O/MeOH/ACN (64:23:13, *v/v/v*) Isocratic program Flow-rate: 1 mL/min Injection volume: 100 μL λexcitation: 364 nm λemission: 440 mm	C18 150 mm × 4.6 mm 5 μm	-	-	0.4–1.3	[74]
Peanuts, almonds and pistachios	AFBI, AFB2, AFG1, AFG2, OTA, FB1, FB2, T-2, HT-2, STE, CIT, DON, ZEN, etc.	QuEChERS	Sample quantity: 5 g Sample extraction:10 mL H_2_O, 10 mL ACN:FA 0.1%; 4 g MgSO4 + 1 g NaCl + 1 g sodium citrate + 0.5 g disodium hydrogen citrate sesquihydrate, centrifugation; d-SPE with EMR-lipid: activation with 5ml H_2_O + 5 mL extrat, centrifugation, 5 mL supernatant + 0.4 g NaCl + 1.6 g MgSO_4_, centrifugation	HPLC-MS	Mobile phase: (A) H_2_O with 0.1% FA, (B) ACN with 0.1% AF Gradient program: 0–5 min 4% B, 5–20 min 100% B, 20–24 min 100% B, 24–28 min 2% B and this latest rate was maintained for 10 min (*t* = 38 min) Flow-rate: 200 nL/min Ionization: ESI source in the positive mode Temperature column: 25 °C Injection volume: 100 nL Ionization: ESI source in the positive mode Capillary voltage: 2.2 kV Collision gas pressure: Vaporizer temperature: 250 °C Sheath gas pressure: Auxiliary gas flow:	Easy-Spray PepMap C18 nano 150 mm × 75 μm 3 μm	-	-	0.05–5	[75]
Raw peanuts and roasted pistachios	AFB1; AFB2; AFG1; AFG2; OTA	QuEChERS	Sample quantity: 2.5 g Sample extration: 10 mL ACN + 10 mL H_2_O with 0.2% FA, rotation for 30 min; 4 g MgSO4 + 1 g NaCl + 1 g sodium citrate + 0.5 g disodium hydrogen citrate sesquihydrate, centrifugation, follow by 2 extraction with 20 mL hexane; d-SPE: supernatant + 150 mg C18 + 900 mg MgSO_4_, centrifugation, wash 2 × with 5 mL ACN	LC—MS/MS	Mobile phase:(A) H_2_O Gradient program: Flow-rate: 0.2 mL/min Temperature column: 30 °C Injection volume: 4 μL Ionization: ESI source in the positive mode Capillary voltage: Collision gas pressure: 25 psi Vaporizer temperature: 250 °C Sheath gas pressure: Auxiliary gas flow: 14 L/min	ODS C18 150 mm × 2.1 mm 5 μm	-	0.05–0.10	0.08–0.30	[76]
Almonds, hazelnuts, peanuts, pistachios, walnuts	AFB1, AFB2, AFG1, AFG2; ZEA	QuEChERS	Sample quantity: 2 g Sample extraction: 10 mL ACN/H_2_O (80:20 *v/v*), rotation for 20 min, 4 g Na_2_SO_4_ + 1 g NaCl, centrifugation; d-SPE: 3 mL supernant + 100 mg C18, centrifugation	UHPLC—MS/MS	Mobile phase: (A) H_2_O with 0.1% FA, (B) ACN with 0.1% FA Gradient program: 25% A increased to 100% in 3.75 min, reduction to 25% A in 6 min (*t* = 7.5 min) Flow-rate: 0.2 mL/min Ionization: ESI source in the negative mode Temperature column: 25 °C Injection volume: 5 μL Ionization: ESI source in the positive mode for AFs Capillary voltage: 3.5 kV Collision gas pressure: 45 psi Vaporizer temperature: 400 °C Sheath gas pressure: Auxiliary gas flow: 11 L/min	C18, 100 mm × 2.1 mm, 1.8 μm	-	-	0.5–1.0	[77]
Almonds, hazelnuts and pistachios	AFBI, AFB2 AFG1, AFG2, OTA, OTB, T-2, HT-2, STE, CIT, DON, ZEN, etc.	QuEChERS	Sample quantity: 1 g Sample extraction: 5 mL H_2_O, 5 mL ACN with 0,1% FA; 0.5 g NaCl + 2 g MgSO4, centrifugation; d-SPE: 1.5 mL supernatant + 50 mg C18, centrifugation	UHPLC—MS	Mobile phase: (A) H_2_O with 0.1% FA, (B)MeOH with 0.1% FA Gradient program: 0% B for 1 min, 95% B for 1.5 min, 75% B for 2.5 min, decrease to 60% in 1 min, back to 0% B in 0.5 min and held for 1.5 min (*t* = 8 min) Flow-rate: 0,4 mL/min Ionization: ESI source in the posiive and negative mode Temperature column: 30 °C Injection volume: 5 μL Ionization: ESI source in the positive and negative mode Capillary voltage: ± 4 kV Collision gas pressure: Vaporizer temperature: 290 °C Sheath gas pressure: 35 psi Auxiliary gas flow:	Luna Omega Polar C18, 50 mm × 2.1 mm, 1.6 μm	-	-	0.2–0.78	[78]

ACN—acetonitrile; ADONs—Sum of 3-acetyl and 15-acetyl-deoxynivalenol; AFB1—Aflatoxin B1; AFB2—Aflatoxin B2; AFG1—Aflatoxin G1; AFG2—Aflatoxin G2; AFM1—Aflatoxin M1; AFM2—Aflatoxin M2; CIT—Citrinin; ELISA—Enzyme-Linked Immunosorbent Assay; ESI—electrospray ionization; FA—formic acid; FB1/FB2—fumonisins; HPLC- FLD—High performance liquid chromatography with Fluorescence Detection; IAC—Immunoaffinity columns; LC—MS—Liquid Chromatography Mass Spectrometry; OTA—Ochratoxin A; OTB– Ochratoxin B; MeOH—methanol; PCD—post column derivatization; PBS—phosphate buffer saline; QuEChERS—Quick, Easy, Cheap, Effective, Rugged and Safe; STE—sterigmatocystin; UHPLC-MS—Ultra-High-Performance Liquid Chromatography tandem Mass Spectrometry; SPME—Solid-Phase Microextraction; T-2/HT-2—Trichothecenes; ZEA—Zearalenone8.1. Sample Preparation.

**Table 2:** Occurrence of mycotoxins in pistachios worldwide.

Reference	Country	Number Samples	Mycotoxin	Nº Positive Samples	% Positive Samples	Average Concentration (μg/kg)	Min-Max (μg/kg)
[24]	Iran	10,068	AFB1	3699	37	5.9	-
			AFs	2852	28	7.3	-
[68]	Algeria	31	AFs	2	6	-	0.4–0.7
			OTA	1	3	170	-
[121]	Spain	70	OTA	2	3	0.228	0.134–0.321
[70]	Iran	32	AFB1	17	53	-	9.5–43.8
			AFB2			-	0.9–9.4
			AFG1			-	n.d.–19.7
			AFG2			-	n.d.–7.1
[122]	Spain	70	AFs	14	20	8.9	n.d.–108
[72]	Saudi Arabia	53	AFS	18	34	16.6	-
		9	AFB1	9		-	1.9–411
			AFB2			-	n.d.–10.7
			AFG1			-	n.d.–4.6
			AFG2			-	n.d.–0.8
[25]	Austria	8	AFs	0	0	-	-
			H-T2	0	-	-	-
			OTA	1	13	<LOQ	-
			T2	0	-	-	-
			ZEA	0	-	-	-
[60]	USA	10	AFB1	2	20	-	0.5–1.2
			AFB2	1	10	0.9	-
			AFG1	1	10	0.5	-
			AFG2	0	-	0.0	-
			DON	0	-	-	-
			FB1	0	-	-	-
			FB2	0	-	-	-
			OTA	3	30	1.4	1.0–6.6
			T2	0	-	-	-
			ZEA	0	-	-	-
[74]	Italy	8	AFB1	4	50	31.9 (median)	8.2–354.5
			AFs		50	33.9 (median)	8.8–387.3
[76]	Malaysia	10	AFB1	4	40	7.10	5.30–10.15
			AFB2	3	30	2.18	1.46–3.47
			AFG1	4	40	2.45	1.90–3.31
			AFG2	2	20	0.86	0.81–0.90
			OTA	0	-	-	-
[123]	Turkey	50	OTA	2	4	0.527	0.198–0.850

AFs—Aflatoxins (AFB1, AFB2, AFG1 and AFG2); AFB1—Aflatoxin B1; AFB2—Aflatoxin B2; AFG1—Aflatoxin G1; AFG2—Aflatoxin G2; FB1 and FB2—Fumonisins; OTA—Ochratoxin A; DON—Desoxynivalenol; T-2/HT-2 –Trichothecenes; ZEA—Zearalenone; LOQ—limit of quantification; n.d.—not defined.

**Table 3:** Summary of studies using decontamination methods to degrade AFB1 in pistachio nuts.

Method	Treatment	Assay Conditions	Reduction AFB1	Reference
Physical	Heat/Roasting	150 °C for 30 min	63%	[143]
	Gamma radiation	10 kGy	68%	[144]
Chemical	Ozonation	0.9 mg/L for 420 min	23%	[145]
	Seed extract *Trachyspermum ammi*	37 °C for 24 h	91%	[146]
	Leaf extract *Adhatoda vasica*	37 °C for 24 h	96%	[147]
	Leaf extract *Corymbia citriodora*	30 °C for 72 h	95%	[148]
	Leaf extract *Ocimum basilicum*	30 °C for 72 h	90%	[149]
Biological	Kefir-grains	30 °C for 6 h	97%	[150]
	*Bacillus subtilis* UTBSP1	35 °C for 5 days	95%	[151]
	*Saccharomyces cerevisiae*	-	40–70%	[152]
Others	Heat + Acidification	15 mL lemon juice 6 g citric acid 120 °C for 1 h	49%	[153]

## Data Availability

Not applicable because the work was based on the literature review.
